# The protein phosphatase 1 regulator NIPP1 is essential for mammalian spermatogenesis

**DOI:** 10.1038/s41598-017-13809-y

**Published:** 2017-10-17

**Authors:** Mónica Ferreira, Shannah Boens, Claudia Winkler, Kathelijne Szekér, Iris Verbinnen, Aleyde Van Eynde, Margarida Fardilha, Mathieu Bollen

**Affiliations:** 1 Laboratory of Biosignaling & Therapeutics, KU Leuven Department of Cellular and Molecular Medicine, University of Leuven, Leuven, Belgium; 2 Institute for Research in Biomedicine-iBiMED, Health Sciences Department, University of Aveiro, Aveiro, Portugal

## Abstract

NIPP1 is one of the major nuclear interactors of protein phosphatase PP1. The deletion of NIPP1 in mice is early embryonic lethal, which has precluded functional studies in adult tissues. Hence, we have generated an inducible NIPP1 knockout model using a tamoxifen-inducible Cre recombinase transgene. The inactivation of the NIPP1 encoding alleles (*Ppp1r8*) in adult mice occurred very efficiently in testis and resulted in a gradual loss of germ cells, culminating in a Sertoli-cell only phenotype. Before the overt development of this phenotype *Ppp1r8* ^*−/−*^ testis showed a decreased proliferation and survival capacity of cells of the spermatogenic lineage. A reduced proliferation was also detected after the tamoxifen-induced removal of NIPP1 from cultured testis slices and isolated germ cells enriched for undifferentiated spermatogonia, hinting at a testis-intrinsic defect. Consistent with the observed phenotype, RNA sequencing identified changes in the transcript levels of cell-cycle and apoptosis regulating genes in NIPP1-depleted testis. We conclude that NIPP1 is essential for mammalian spermatogenesis because it is indispensable for the proliferation and survival of progenitor germ cells, including (un)differentiated spermatogonia.

## Introduction

Spermatogenesis is a highly dynamic process that requires the coordinated proliferation and differentiation of male germ cells^1–4^. Spermatogonial stem cells (SSCs) represent the most primordial germ cells in adult testis; they divide cyclically to generate more stem cells (self-renewal) or progeny committed to differentiate into primary spermatocytes. The latter cells undergo a first meiotic division to form secondary spermatocytes, which rapidly enter the second meiotic division to produce spermatids. Finally, spermatids develop into spermatozoa during a differentiation process that involves major morphological changes. Spermatogenesis takes place in seminiferous tubules, which are surrounded by interstitial cells, including the testosterone-producing Leydig cells. The seminiferous tubules contain the full complement of germ cells, which gradually move from the periphery to the central lumen during maturation. The total number of germ cells in the seminiferous tubules is strictly controlled and mainly determined by coordination of cell proliferation and apoptosis^5,6^. The seminiferous tubules also contain Sertoli cells, which provide structural and metabolic support to the germ cells^7,8^.

Germ-cell proliferation and differentiation is tightly regulated by reversible protein phosphorylation^9^. While the role of various protein kinases has already been firmly established very little is known about the counteracting phosphatases. Protein phosphatase 1 (PP1) catalyzes a major fraction of phospho-serine/threonine dephosphorylation reactions and it does so in a highly specific manner because it forms stable complexes with a host of PP1-interacting proteins that determine the activity and selectivity of the phosphatase^10^. Nuclear Inhibitor of PP1 (NIPP1; 351 residues in mouse), encoded by *Ppp1r8*, is one of the major nuclear interactors of PP1 and is expressed in nearly all eukaryotic cells. The N-terminal third of NIPP1 largely consists of a well-structured ForkHead-Associated (FHA) domain, while the remainder of the protein is intrinsically disordered but becomes partially structured upon binding to PP1^11^. The FHA domain of NIPP1 mediates the recruitment of a subset of phosphoproteins, including the pre-mRNA splicing factors SAP155 and CDC5L^12,13^, protein kinase MELK^14^ and the methyltransferase EZH2^15^, for regulated dephosphorylation by associated PP1. Consistent with the diversity of its FHA ligands, NIPP1 has been implicated in both transcription^16–18^ and pre-mRNA splicing^19^, and has established contributions to cell proliferation and differentiation^15,20–23^.

The global deletion of NIPP1 in mice is early embryonic lethal^23^. NIPP1^*−/−*^ embryos show a reduced cell proliferation potential and die at the gastrulation stage. In contrast, the selective removal of NIPP1 from liver epithelial cells is not lethal, but results in an increased proliferation of biliary epithelial cells in the adult liver, including progenitor cells^22^. To further explore the exact function of NIPP1 in adult tissues we have generated an inducible knockout model. Here, we describe the testicular phenotype of the tamoxifen-induced deletion of NIPP1 in adult mice. Strikingly, the postnatal deletion of NIPP1 from testis results in the progressive loss of germ cells, leading to testicular hypoplasia within a few months. This correlates with a reduced proliferation and survival capacity of germ cells, including (un)differentiated spermatogonia. Our results demonstrate that NIPP1 is essential for the maintenance of the male germline and sustained spermatogenesis.

## Results

### Generation of an inducible NIPP1-knockout model

**Figure 1:**
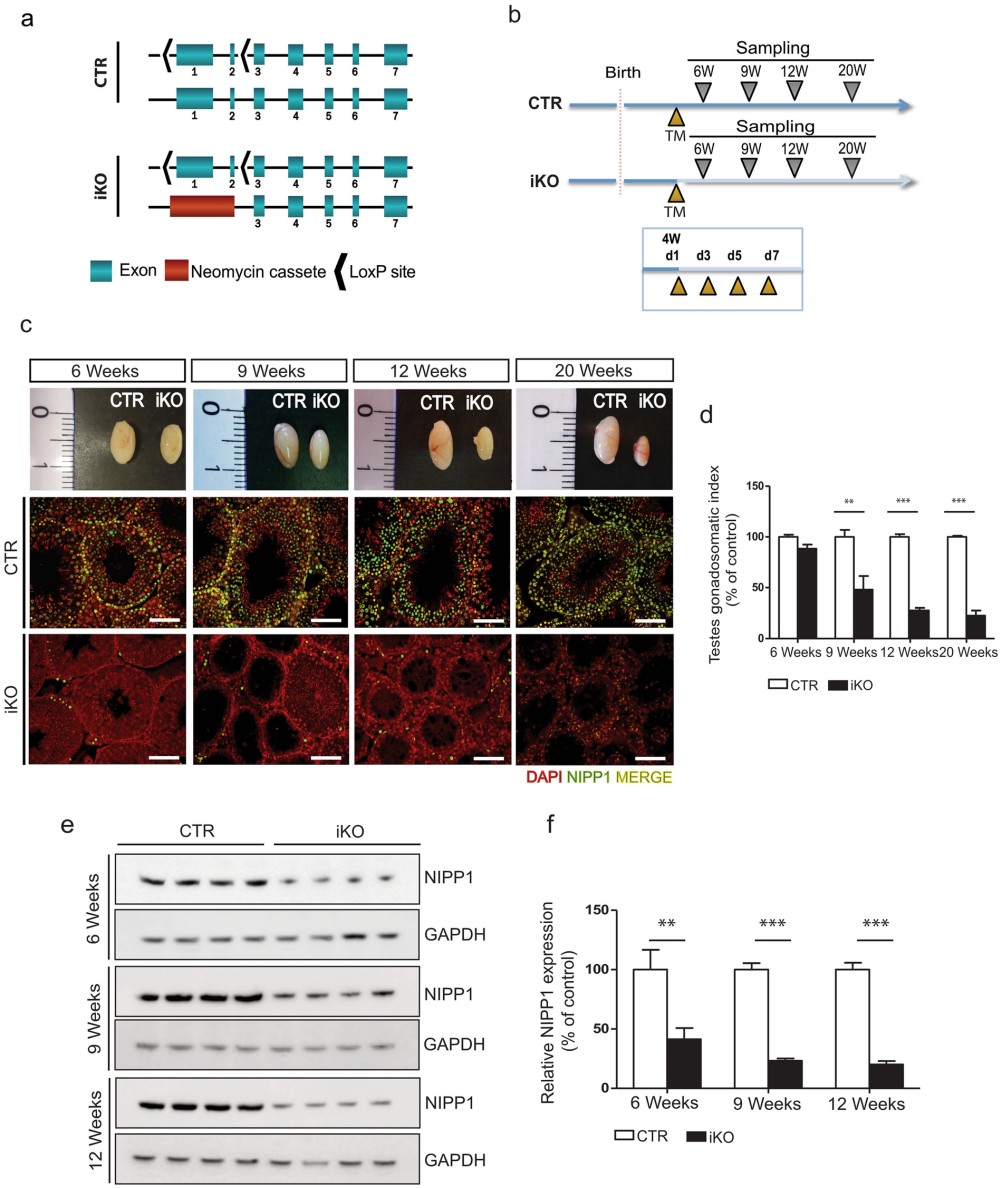
The postnatal inactivation of *Ppp1r8* in adult mice causes a reduced testis size. (**a**) *Ppp1r8* alleles in control (CTR) and inducible NIPP1 knockout (iKO) mice. The exon numbers are indicated. (**b**) CTR and iKO mice express CRE-ERT2 recombinase under control of the UBC promoter (*Ubc*-*Cre*-ERT2). Scheme of the 4 consecutive (every 2 days starting at the age of 4 weeks) intraperitoneal tamoxifen (TM) injections at the age of 4 weeks (W) and subsequent testes sampling. d, days. (**c**) Macroscopic view of tamoxifen-injected testes from CTR and iKO mice at the age of 6, 9, 12 and 20 weeks (upper panels). The lower panels show DAPI (red) and NIPP1 (green) stainings of testis sections from tamoxifen-treated CTRs and iKOs at the indicated ages. Scale bar, 50 μm. (**d**) Testes gonadosomatic index, as determined by the percentage of total testis weight (g) over the body weight (g), of 6, 9, 12 and 20 weeks-old adult tamoxifen-treated CTR and iKO mice. (**e**,**f**) The level of NIPP1 in total testis extracts from tamoxifen-injected CTR and iKO mice was visualized by immunoblotting (**e**) and quantified (**f**). GAPDH was used as a loading control. Data are represented as means ± SEM (n = 4). **p < *0*.01; ***p < *0*.001. Uncropped blots are presented in Supplementary Fig. 8.

To study the postnatal functions of NIPP1 we generated an *in vivo* model for the inducible inactivation of *Ppp1r8*. Mice with floxed *Ppp1r8* alleles (*Ppp1r8* ^*fl/fl*^) were crossed with transgenic mice expressing tamoxifen-activated CRE-ERT2 recombinase under control of the Ubiquitin C (UBC) promotor (Supplementary Fig. 1a–c)^24^. Offspring with the *Ubc-Cre-ERT2* ^+/−^; *Ppp1r8* ^*fl/+*^ genotype was crossed with heterozygous mice (*Ppp1r8* ^+/−^). The resulting *Ubc-Cre-ERT2* ^+/−^; *Ppp1r8* ^*fl/−*^ mice were used as tamoxifen-inducible NIPP1 knockouts or iKOs (Fig. 1a, Supplementary Fig. 1a–c). Since the deletion of one *Ppp1r8* allele does not affect the expression level of NIPP1^23^, the heterozygous *Ubc-Cre-ERT2* ^+/−^; *Ppp1r8* ^*fl/+*^ mice were used as controls (CTRs). The adopted knockout strategy avoids phenotypic artefacts induced by CRE recombinase because one *Ppp1r8* allele is inactivated by tamoxifen-induced recombination in both the CTRs and iKOs^25^.

Following 4 consecutive tamoxifen injections at the age of 4 weeks, the CTR and iKO mice were sacrificed 2–16 weeks later (Fig. 1b). The iKO mice appeared healthy and did not display any overt phenotype, except for a slightly lower body weight at 12 weeks (Supplementary Fig. 1d). In fact, the only macroscopic phenotype after dissection was a gradually developing smaller testis size in the male iKOs (Fig. 1c and d). This correlated with a loss of NIPP1 from the testis, as shown by both immunohistochemistry (Fig. 1c) and immunoblotting (Fig. 1e and f). At the age of 6 weeks, the NIPP1 protein level in the iKO testis was already reduced to about 40% of that in CTR testis, and further decreased to about 20% at 9–12 weeks. The remaining NIPP1 in the iKOs was mainly expressed in interstitial cells (Fig. 1c). We confirmed that the expression level of NIPP1 in testis from wild-type and *Ppp1r8* ^+/−^ mice was the same (Supplementary Fig. 1e and f), justifying the use of heterozygotes as controls. Remarkably, in the accessory sex glands (seminal vesicles and agglutination glands) and epididymis, a depletion of NIPP1 was only detected in limited histological areas and this had no effect on the histological organization and size of these glands (Supplementary Fig. 2). This suggests that the tamoxifen-induced NIPP1 deletion is variable between tissues and even within tissues, in accordance with previous findings^26^.

### The postnatal deletion of NIPP1 leads to a loss of male germ cells

**Figure 2:**
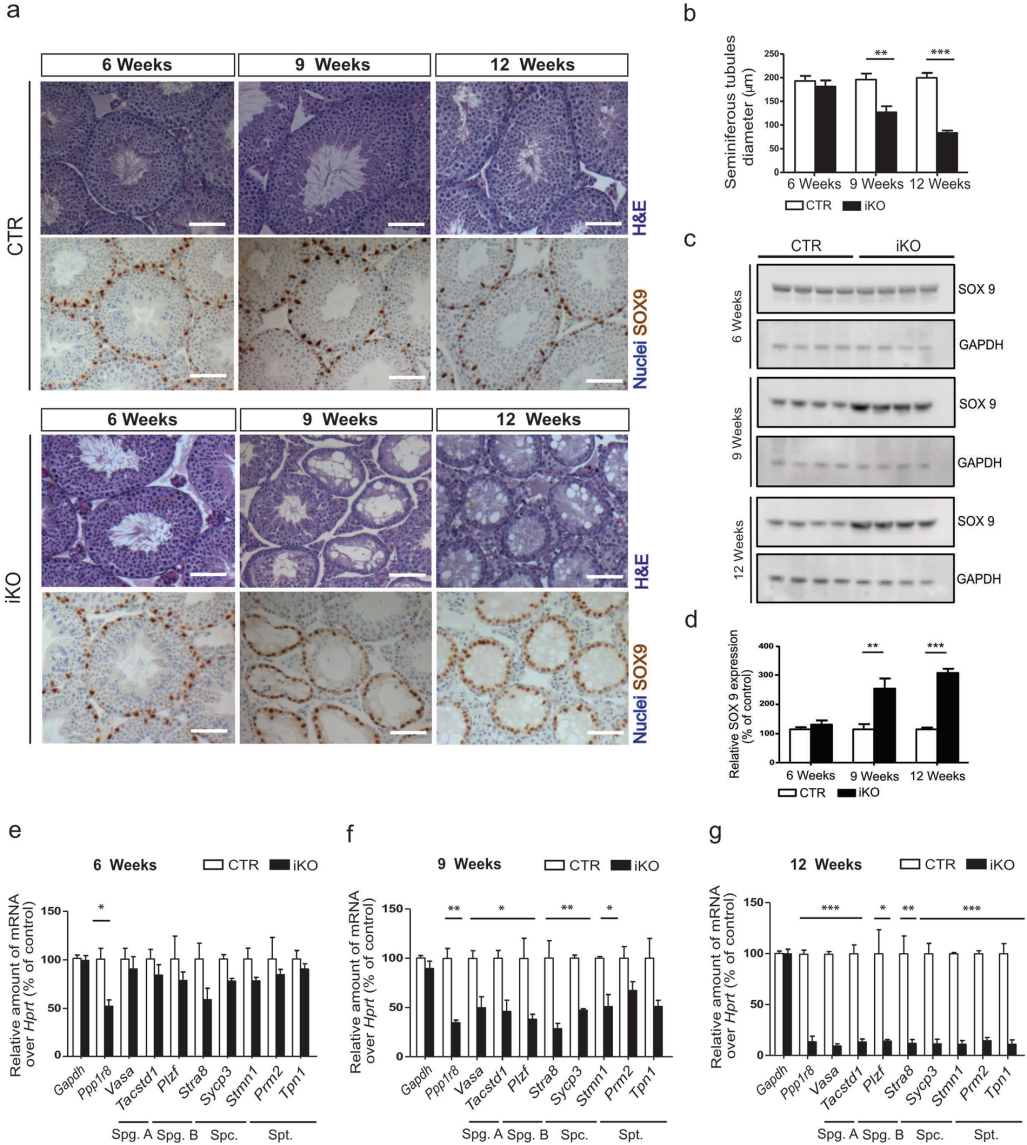
The postnatal deletion of NIPP1 in adult mice leads to a loss of germ cells. (**a**) Testis sections of tamoxifen-injected CTR and iKO mice at the indicated ages were Hematoxylin-Eosin (H&E) stained or immunostained for the Sertoli-cell marker SOX9 (chromogenic DAB detection), with a Hematoxylin counterstain for the visualization of nuclei. Scale bar, 50 μm. (**b**) Quantification of cross-sectional diameter of the seminiferous tubules. (**c**,**d**) The level of SOX9 in total testis extracts from tamoxifen-injected CTR and iKO mice at the age of 6, 9 and 12 weeks was visualized by immunoblotting (**c**) and quantified (**d**). GAPDH was used as a loading control. (**e–g**) qRT- PCR analysis of the indicated genes, including markers for the different types of germ cells, at the indicated ages. *Hprt* was used as housekeeping gene for normalization. Spg. A, spermatogonia A; Spg. B, spermatogonia B; Spc., spermatocytes; Spt., spermatids. Data are represented as means ± SEM (n ≥ 3). *p < 0.05; **p < 0.01; ***p < 0.001.

In adult mouse testis NIPP1 is expressed in both germ cells and somatic cells, including Sertoli cells (Supplementary Fig. 3). Immunostaining revealed NIPP1 expression in all types of germ cells, except in elongated terminally differentiated spermatids. At the age of 6 weeks, which is two weeks after the administration of tamoxifen, NIPP1 was largely removed from both germ cells and Sertoli cells (Fig. 1c and Supplementary 3c). This incited us to explore the fate of both cell types in the NIPP1 iKOs. H&E stainings revealed that the diameter of the seminiferous tubules gradually decreased due to a loss of germ cells (Fig. 2a and b). In testis of 9 weeks, i.e. 5 weeks after the administration of tamoxifen, the seminiferous tubules already showed a clear decrease in the number of germ cells (Fig. 2a). The most severely affected tubules showed gross vacuolization and only contained few germ cells. By the age of 12 weeks all seminiferous tubules were histologically agametic and only contained Sertoli cells. However, the number of Sertoli cells per seminiferous tubule (SOX9 staining) was not affected by the deletion of NIPP1 (Fig. 2a and Supplementary Fig. 3d). These data imply that the testis from tamoxifen-treated iKO mice at 9 and 12 weeks are relatively enriched for Sertoli cells, as independently confirmed by SOX9 immunoblotting (Fig. 2c and d). At 20 weeks the Sertoli-cell only phenotype was still maintained, indicating that no new cycles of spermatogenesis had been started after week 9–12 (not shown). Quantitative RT-PCR for the global germ-cell marker *Vasa* ^27,28^ and stage-specific germ cell markers for spermatogonia (*Tacstd1*, *Plzf*, *Stra8*)^29–33^, spermatocytes *(Sycp3*, *Stmn1)* ^34,35^ and spermatids (*Prm1*, *Tpn1*)^36,37^, confirmed the progressive loss of these types of germ cells in the iKOs (Fig. 2e–g). Hence, the postnatal deletion of NIPP1 from testis results in the gradual loss of germ cells and culminates within a few cycles of spermatogenesis in a Sertoli-cell only phenotype.

### The postnatal deletion of NIPP1 reduces the proliferation and survival of germ cells

**Figure 3:**
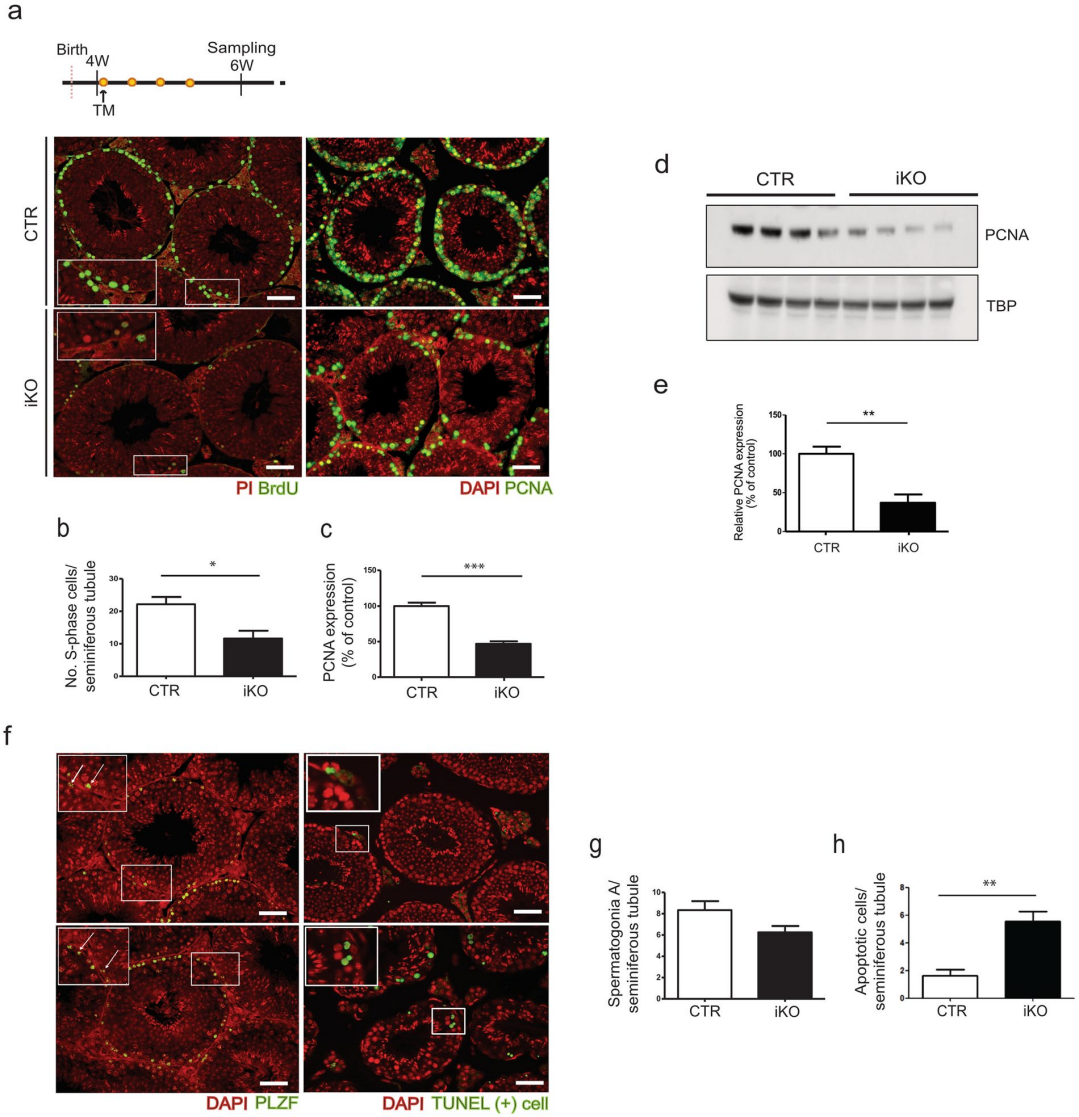
*Ppp1r8* ^*−/−*^ testes show a reduced proliferation and survival of germ cells. (**a**) Testis sections of tamoxifen-treated mice of 6 weeks were immunostained for incorporated BrdU and the proliferation marker PCNA. Nuclei were visualized with propidium iodide (PI) or DAPI, as indicated. Scale bar, 50 μm. (**b–c**) Quantifications of the stainings as shown in panel (**a**) and performed as described in Materials and Methods. (**d–e**) The level of PCNA in total testis extracts from tamoxifen-injected CTR and iKO mice at the age of 6 weeks was visualized by immunoblotting (**d**) and quantified (**e**). TBP was used as a loading control. (**f**) Testis sections of tamoxifen-treated mice of 6 weeks were immunostained for the spermatogonial marker PLZF, and stained by a fluorochrome-based TUNEL assay to visualize apoptosis. Nuclei were visualized by DAPI. Scale bar, 50 μm. (**g**,**h**) Quantifications of the stainings as shown in panel (**f**) and performed as described in Materials and Methods. All bar data are means** ± **SEM (n = 4). *p < 0.05; **p < 0.01; ***p < 0.001.

We subsequently examined whether the testis phenotype in the iKOs can be explained by a reduced proliferation and/or an increased apoptosis of germ cells. For these experiments, we used tamoxifen-treated mice of 6 weeks, when a testis phenotype is not yet histologically apparent (Fig. 2). Both BrdU (5′-bromo-2′-deoxyuridine) incorporation assays (Fig. 3a and b) and PCNA (proliferating cell nuclear antigen) stainings (Fig. 3a and c) disclosed a twofold lower proliferation rate of spermatogonia and (pre)leptotene spermatocytes in the iKOs. These conclusions were validated by immunoblotting for PCNA (Fig. 3d and e) and immunostainings for cyclin D2 and histone H3 phosphorylation at Ser10 (H3S10P), which are markers for G1 and mitotic cells, respectively (Supplementary Fig. 4a–c). Finally, we confirmed that no significant loss of germ cells was detected yet in the iKOs at 6 weeks, as demonstrated by qRT-PCR data (Fig. 2e) and immunostainings for the spermatogonia marker PLZF (promyelocytic leukemia zinc finger)^29,30^ (Fig. 3f and g).

TUNEL assays revealed a 3-fold increased level of apoptosis in germ cells from 7 weeks-old iKO mice (Fig. 3f and h). Apoptosis was particularly increased in germ cells from the basal layers of the seminiferous epithelium, comprising spermatogonia and early meiotic spermatocytes. Stainings for the senescence marker p16^INK4a^ (Supplementary Fig. 4a and d) and for fibrosis (Supplementary Fig. 4e) did not disclose differences between the CTRs and iKOs. Also, both groups contained similar proportions of tubules at different stages of development (Supplementary Fig. 4f). Collectively, our data indicate that the gradual loss of male germ cells after the deletion of NIPP1 is explained by their reduced proliferation and increased apoptosis rather than by the induction of senescence or fibrosis. We also examined whether the loss of germ cells in the iKOs was associated with a destabilization of NIPP1 ligands. However, immunoblotting of testicular lysates of mice of 6 weeks did not disclose a change in the level of three major protein interactors of NIPP1 in the iKOs, i.e. protein phosphatase PP1 or the splicing factors CDC5L and SAP155 (Supplementary Fig. 5).

### Germ-cell proliferation is also reduced after the deletion of NIPP1 in cultured testis slices

**Figure 4:**
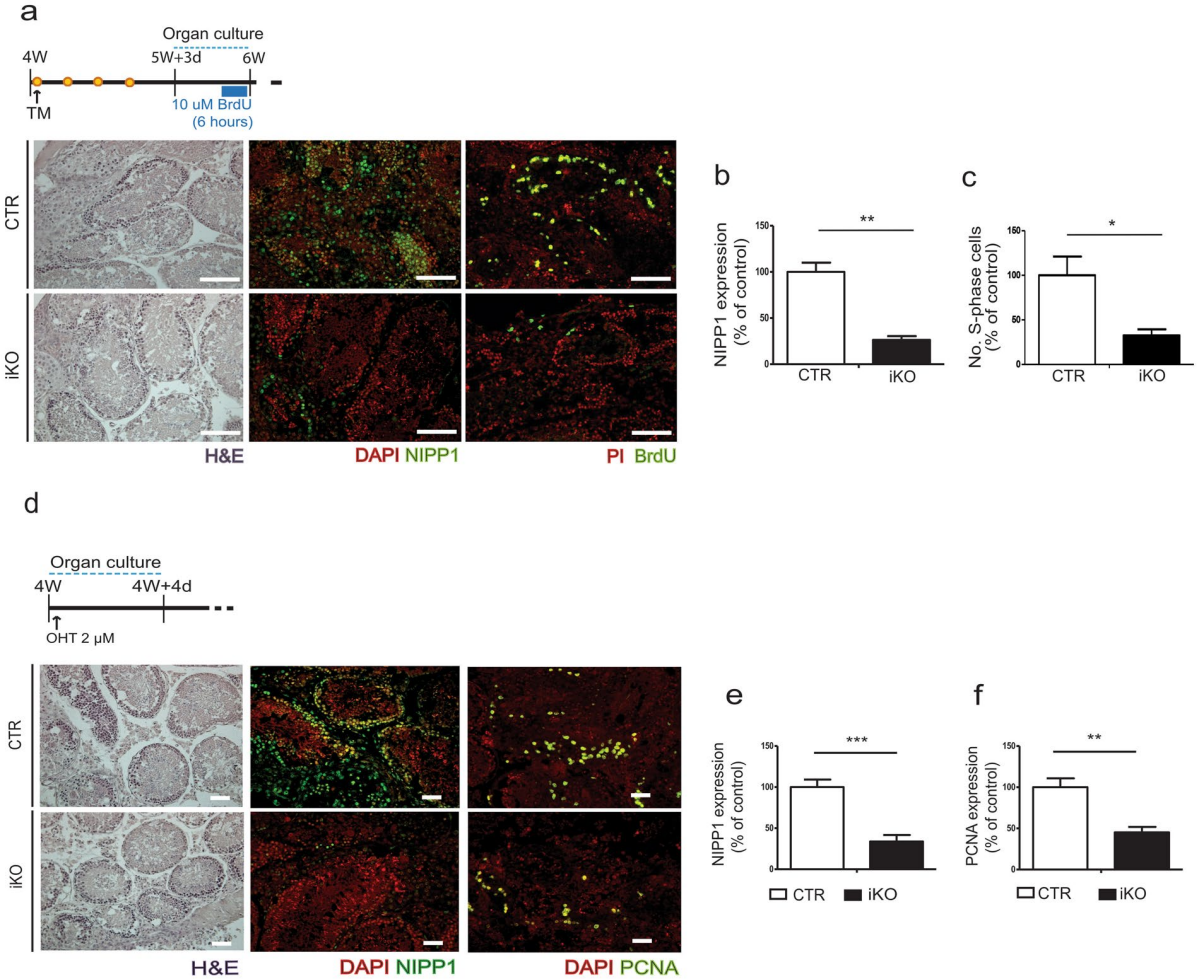
The deletion of NIPP1 leads to a reduced proliferation of germ cells in cultured testis slices. (**a**) Testis from tamoxifen-treated CTR and iKO mice were isolated at 5 weeks and 3 days, and cultured for 4 days. After incubation for 6 h with BrdU, testis sections were H&E stained and immunostained for NIPP1 and incorporated BrdU. Nuclei were visualized with propidium iodide (PI) or DAPI as indicated. Scale bar, 50 µm. (**b**,**c**) Quantification of stainings for NIPP1 (**b**) and incorporated BrdU (**c**) as shown in panel (a). (**d**) Organ culture of testis slices that were isolated from non-treated CTRs and iKOs of 4 weeks. (Z)-4-Hydroxytamoxifen (4-OHT) was added to the slices for 96 hours to delete the floxed *Ppp1r8* allele. Subsequently, testis sections were H&E stained, and immunostained for NIPP1 and PCNA. Nuclei were visualized by DAPI. Scale bar, 50 μm. (**e**,**f**) Quantification of stainings as shown in panel (d). All data in this figure are represented as means ± SEM (n = 4). *p < 0.05; **p < 0.01; ***p < 0.001.

To further explore the origin of the loss of germ cells in the iKOs, we have subsequently investigated whether this could be an indirect effect caused by the deletion of NIPP1 from peripheral tissues. For example, it could be argued that the deletion of NIPP1 from the brain interferes with the level of circulating testosterone, which is required for germ-cell survival^38^. However, the expression of NIPP1 in the iKOs was only marginally affected in the brain cortex and hypothalamus, and only reduced in a scattered fashion in the cerebellum (Supplementary Fig. 6a-d). Also, the levels of circulating testosterone at 9 weeks were similar in the CTRs and iKOs and there was no correlation between testosterone levels and testis weight (Supplementary Fig. 6e and f). To further differentiate between intrinsic and extrinsic defects, we aimed to generate a testis-specific NIPP1 mouse KO model by recombination with Cre recombinase under control of the gonocyte-specific *Vasa* promotor (Supplementary Fig. 6g and h). However, the CRE-recombinase was expressed precociously, resulting in the global deletion of NIPP1, as has been reported for other Cre strains^39^. As an alternative approach to determine whether the testis phenotype was mediated by the loss of NIPP1 from peripheral tissues, we performed organ cultures using testis that were isolated from tamoxifen-treated mice of 6 weeks (Fig. 4a), or testis isolated at the age of 4 weeks and treated *in vitro* with hydroxytamoxifen (Fig. 4d). Under *in vitro* outgrowing conditions, BrdU incorporation (Fig. 4a–c) and the level of PCNA (Fig. 4d–f) were severely decreased in the iKOs, demonstrating that the observed phenotype is a testis-intrinsic defect.

### NIPP1-depleted spermatogonia have a reduced proliferation capacity

**Figure 5:**
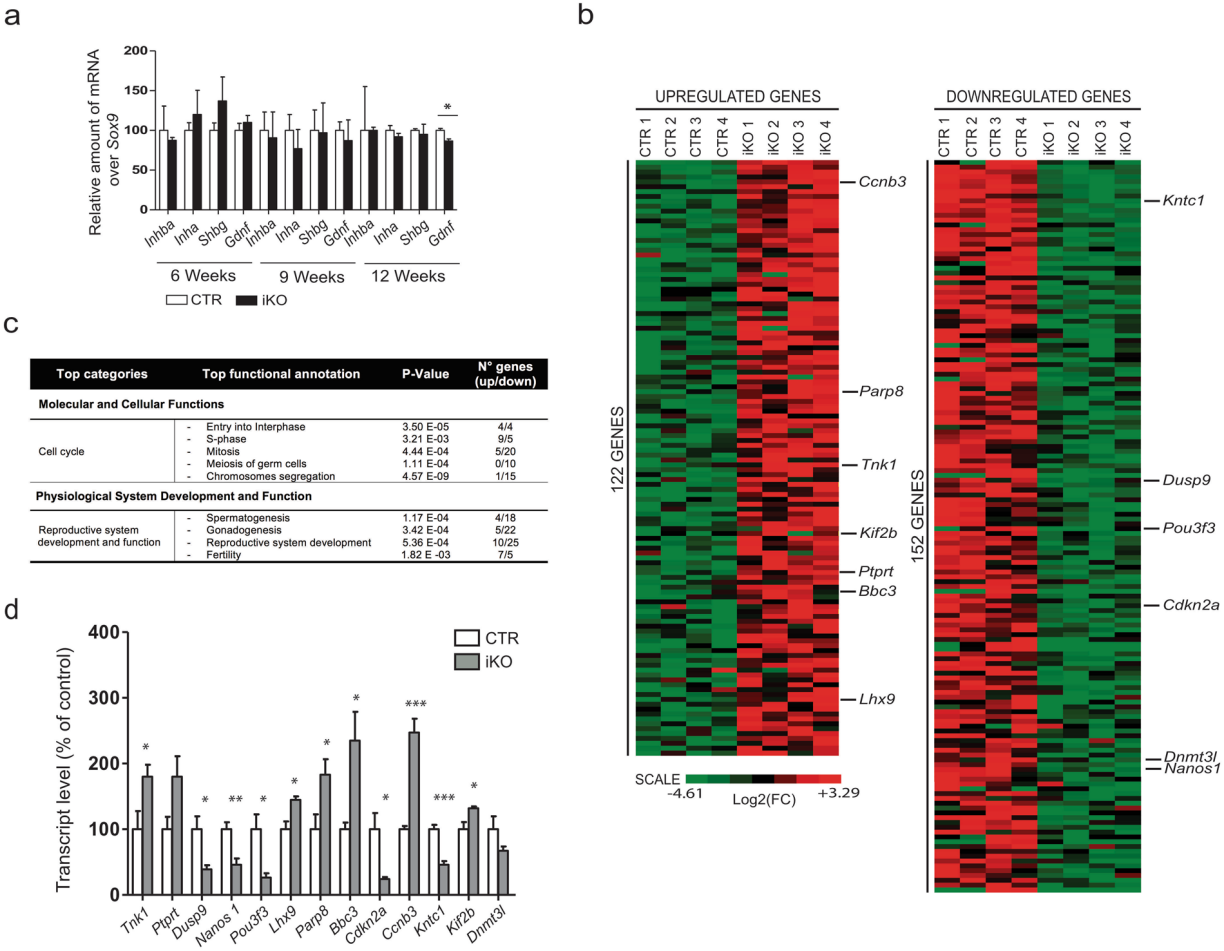
The loss of NIPP1 is associated with the deregulation of cell-cycle and survival-related genes. (**a**) qRT-PCR analysis of genes encoding factors secreted by Sertoli cells, at the indicated ages. *Sox9* was used for normalization. (**b**) Comparative RNA-sequencing profiling in testis of tamoxifen-treated mice of 6 weeks (n = 4). The figure shows the heat map of the significantly up- and downregulated genes. (**c**) Table showing the results of an IPA analysis of the genes that were significantly (FDR p < 0.01) affected by the deletion of NIPP1 in testis of mice of 6 weeks. (**d**) qRT-PCR analysis showing differences at the level of the indicated transcripts between testis of CTRs and iKOs of 6 weeks. The depicted genes encode proteins with the same name except for *Bbc3* and *Cdkn2a*, which encode PUMA and p16/INK4a, respectively. Data are represented as means ± SEM (n = 4). *p < 0.05; **p < 0.01; ***p < 0.001.

Since our data suggested that the observed testis phenotype in the iKOs represents an intrinsic defect, we proceeded to examine the functionality of Sertoli cells, as they are required for the maintenance of spermatogenesis^40^. qRT-PCR revealed that the expression of factors secreted by Sertoli cells, including inhibins (*Inhba* and *Inha*) and the paracrine factor *Gdnf* that promotes spermatogonial stem cell self-renewal (Fig. 5a), were the same in the CTRs and iKOs. Only a minor decrease (~10%) of *Gdnf* was detected at 12 weeks, when all germ cells were already lost in the iKOs. As a complementary approach, we performed RNA sequencing on whole testis isolated from tamoxifen-treated mice of 6 weeks. Data analysis (FDR, <0.05; cut-off, 1.5 fold change) identified 122 upregulated and 152 downregulated genes in the iKOs, but did not disclose changed transcript levels of factors secreted by the Sertoli cells (Fig. 5b, Supplementary Table 1). An Ingenuity Pathway Analysis (IPA) (FDR,* < *0.01; no cut-off) revealed that differentially altered genes in the iKOs were mostly enriched for ‘Cell cycle’ in the IPA category ‘Molecular and Cellular functions’ (Fig. 5c). Among the cell-cycle classified genes ~75% were downregulated, including *Kntc1*, *AurkA*, *Cenpt*, *Ccnb2*, *Nusap1* and *Fancd2*. In the IPA category ‘Physiological System Development and Function‘, most of the deregulated genes were assigned to ‘Reproductive system development and function’, including *Tnk1*, *Nanos1*, *Pou3f3* and *Lhx9*. Finally, we also identified pro-apoptotic genes *(Parp8*, *Bbc3*, *Ccnb3*) that were upregulated in the iKOs. In general, these altered expression profiles are consistent with the observed decreased proliferation and survival potential of testicular cells in the iKOs. Key RNA sequencing data were largely confirmed by qRT-PCR (Fig. 5d). Taken together, these data suggested that the proliferation and survival of testicular cells is decreased in the iKOs, but this is not the consequence of an altered Sertoli-cell secretome.

**Figure 6:**
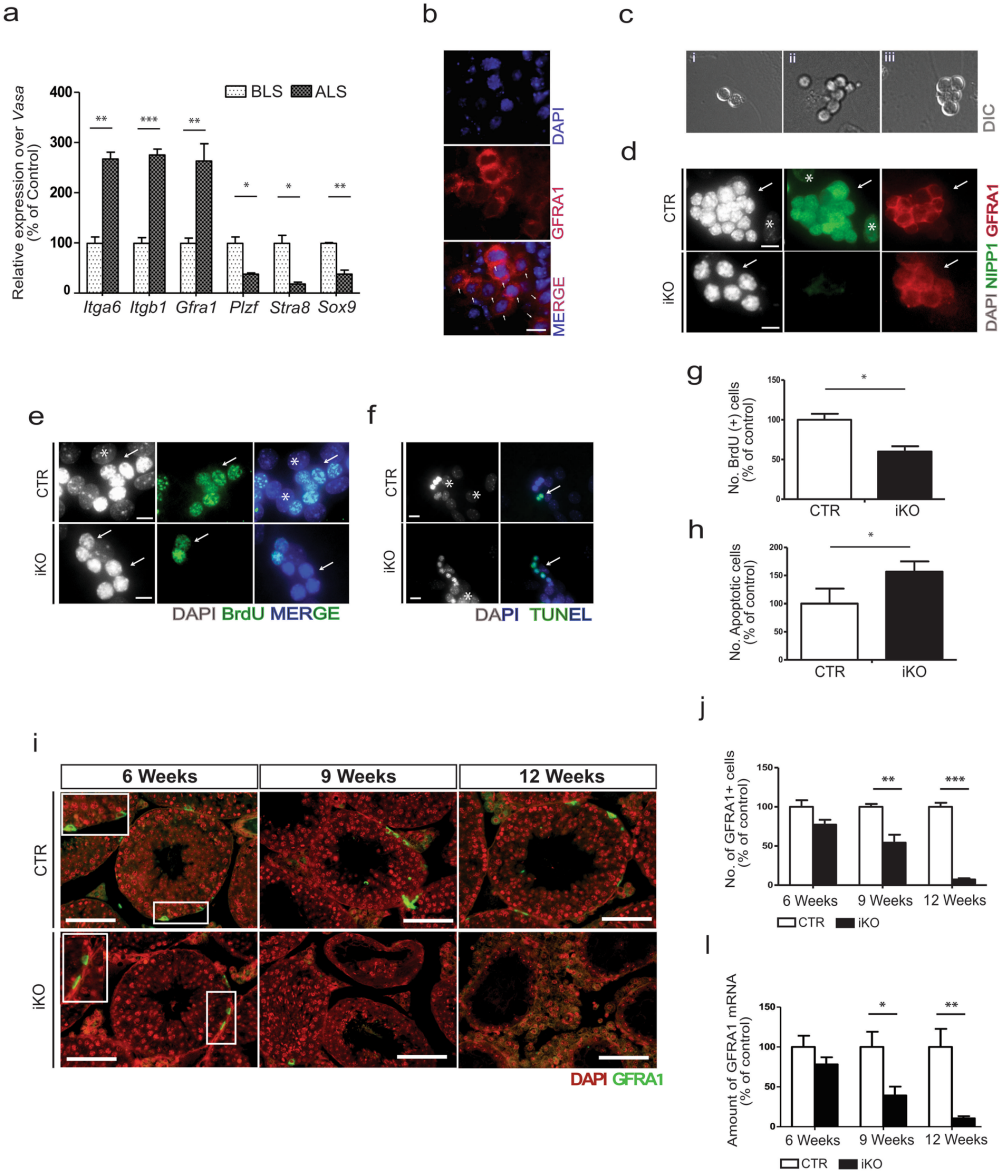
NIPP1 depletion results in the hypoproliferation and progressive loss of (un)differentiated spermatogonia. (**a**) Testicular cells were isolated from 12 days old (P12) non-treated testis and enriched for undifferentiated spermatogonia using the laminin selection method. The graph shows qRT-PCR analysis of the indicated stage-specific markers for undifferentiated spermatogonia (*Gfra1*, *Itgb1 and Itga6*), spermatogonia (*Plzf* and *Stra8*) and Sertoli cells (*Sox9*), before and after laminin selection. The germ-cell specific marker *Vasa* was used for normalization. BLS, before laminin selection; ALS, after laminin selection. (**b**) Cells obtained by laminin selection were stained for DNA (DAPI) and GFRA1 (red). Arrows indicate GFRA1^+^ cells. Representative images are shown. (**c**) Cells that were acquired after the laminin selection as described in panel (**a**) were cultured for 72 h. Representative images generated by differential interference contrast (DIC) of paired cells (left), aligned cells (middle) or colony-forming cells (right) (**d**) Colonies from the CTR and iKO cells were treated for 72 h with 1 µM of 4-OHT and subsequently stained for DNA (DAPI), NIPP1 (green) and GFRA1 (red). Arrows indicate GFRA1^+^ colonies formed after 96 h. *Mouse embryonic fibroblast (MEF) nucleus. Scale bar, 10 μm. (**e–h**) Hydroxytamoxifen-treated GFRA1-enriched cell cultures of CTR and iKO cells, as described in panel (d), were stained for DNA (DAPI), incorporated BrdU (**e**) and apoptosis by TUNEL assay (**f**). Representative images are shown. Arrows indicate GFRA1^+^ colonies. *Mouse embryonic fibroblast (MEF) nucleus. Scale bar, 10 μm. Quantification (n = 3) of stainings for incorporated BrdU (**g**) and apoptotic cells (**h**), as shown in panels (e) and (f), respectively. *Mouse embryonic fibroblast (MEF) nucleus. Scale bar, 10 μm. (**i**) Testis sections of tamoxifen-treated CTR and iKO mice at the indicated ages were stained for DNA (DAPI) and the undifferentiated spermatogonia marker GFRA1. Scale bar, 50 μm. (**j**) Quantification (n = 4) of the relative number of GFRA1^+^ cells per seminiferous tubule. (**l**) qRT-PCR analysis (n = 4) of the *Gfra1* transcript at the indicated ages using *Hprt* as housekeeping gene for normalization. All data in this figure are represented as means ± SEM. *p < 0.05; **p < 0.01; ***p < 0.001.

Given the loss of the male germline in *Ppp1r8* ^*−/−*^ testis, we hypothesized that the progenitor germ cells, comprising (un)differentiated spermatogonia, have a reduced proliferation capacity after the deletion of NIPP1. Testis from 12 days old mice do not yet contain the full complement of germ cells that are present in adult testis but have a higher proportion of spermatogonia, including SSCs. First, we isolated testicular cells from non-treated mice of 12 days old and enriched these cells for undifferentiated spermatogonia by laminin selection^41^, as validated by qRT-PCR expression analysis (Fig. 6a). Indeed, the relative expression of the undifferentiated spermatogonial markers *Itga6* ^41–43^, *Itgb1* ^41–43^ and *Gfra1* ^44–47^ was about 3-fold increased after laminin selection, while other stage-specific germ cell markers (*Plzf* and *Stra8*) and a Sertoli marker (*Sox9*) were decreased. Also, ~70% of laminin-selected cells were GFRA1 positive (Fig. 6b). When the isolated cells were cultured *in vitro* using SSC medium that contains GDFN to permit SSC proliferation independent of Sertoli cells, this resulted in the formation of colonies as described^41^ (Fig. 6c). Subsequently, GFRA1-enriched testicular cells from both CTRs and iKOs were cultured and treated with hydroxytamoxifen for 72 h to delete NIPP1 in iKO cells. Immunostaining for NIPP1 and GFRA1 confirmed the efficient removal of NIPP1 in GFRA^+^ cells (Fig. 6d). The 4-OHT-induced deletion of NIPP1 in GFRA1-enriched cell cultures reduced their proliferation by some 40%, as demonstrated by BrdU incorporation assays (Fig. 6e and g), and increased the number of apoptopic cells, as shown by TUNEL assays (Fig. 6f and h). Consistent with these data, stainings for GFRA1 revealed that the number of GFRA1 cells was reduced by ≈50% and ≈90% in tamoxifen-treated iKOs of 9 and 12 weeks, respectively (Fig. 6i and j). qRT-PCR analysis confirmed the gradual loss of GFRA1^+^ cells in tamoxifen-treated iKO (Fig. 6 l).

Finally, we also investigated the testis of tamoxifen-treated neonates of 7 days. At this age, the seminiferous tubules only contain a heterogeneous pool of gonocyte-derived (un)differentiated spermatogonia and Sertoli-cells (Supplementary Fig. 7a). In the neonatal iKOs NIPP1 was successfully deleted while the number of Sertoli cells (SOX9) was not significantly affected (Supplementary Fig. 7a–d). In contrast, the number of GFRA1^+^ cells was decreased by about 80%, showing that undifferentiated GFRA1^+^ spermatogonia are also depleted during the first wave of spermatogenesis when NIPP1 is absent (Supplementary Fig. 7a and b).

## Discussion

The inactivation of both *Ppp1r8* alleles in mice is early embryonic lethal^23^. This prompted us to develop an inducible knockout model to study postnatal functions of NIPP1. The only macroscopic consequence of the deletion of NIPP1 in adult mice was a reduced testis size (Fig. 1). However, this does not preclude key functions for NIPP1 elsewhere as inactivation of the floxed *Ppp1r8* allele by *Ubc*-Cre-ERT2 recombination was incomplete or patchy in all other examined tissues. Tamoxifen-induced *Ubc*-Cre-ERT2 recombination is indeed known to be tissue-dependent^25,48^. This has been explained by the unequal tissue distribution of injected tamoxifen, distinct chromatin compaction states of the targeted gene which determine its accessibility for Cre-mediated recombination and/or divergent Cre-ERT2 expression levels^26,49^. Nonetheless, *Ubc*-Cre-ERT2 recombination was very efficient in testis and the postnatal deletion of NIPP1 led to a gradual loss of sperm cells, culminating in a Sertoli-only phenotype (Fig. 2).

We have explored the events leading up to the testis phenotype in the iKOs. For this purpose, we mainly analyzed testes of mice of 6 weeks, i.e. two weeks after the administration of tamoxifen, when the seminiferous tubules still appeared normal and the number of germ cells had not yet significantly decreased. Strikingly, at this age we already observed in the tamoxifen-treated iKOs a reduced proliferation and increased apoptosis of spermatogonia and early meiotic spermatocytes (Fig. 3). The reduced proliferation of germ cells was clearly a testis-intrinsic defect because this phenotype was also provoked by the hydroxytamoxifen-induced deletion of NIPP1 in cultured testis slices (Fig. 4). We speculate that the hypoproliferation defect is germ-cell autonomous because GFRA1-enriched cells showed *in vitro* a reduced proliferation after the deletion of NIPP1 (Fig. 6). Consistent with a germ-cell intrinsic defect, the secretome of the germ-cell supporting Sertoli cells (Fig. 5) and the release of testosterone by the Leydig cells (Supplementary Fig. 6) were not affected in the iKOs. It should be noted that the proposed contribution of NIPP1 to germ-cell proliferation and survival does not exclude additional functions for NIPP1 in testis, for example in meiotic germ cells.

The GFRA1-enriched cell population that we isolated comprises various subpopulations of spermatogonia, including spermatogonial stem cells (SCCs). Since GFRA1-enriched cells showed a reduced proliferation after the deletion of NIPP1 (Fig. 6), this suggests that NIPP1 may be required for the proliferation/selfrenewal of SSCs. This hypothesis can be further explored using SSC transplantation assays^50,51^. Intriguingly, while the loss of NIPP1 in both testis (this work) and early embryo’s^23^ is associated with impaired cell proliferation, the selective depletion of NIPP1 in liver epithelial cells results in the hyperproliferation of biliary epithelial cells (BECs), including liver progenitor cells. Thus, NIPP1 emerges as a key regulator of cell proliferation, but its exact role is clearly tissue and context dependent.

Since NIPP1 controls the dephosphorylation of FHA ligands by associated PP1 (see Introduction), we hypothesize that the proliferation defect in NIPP1-depleted testis stems from an altered phosphorylation status of FHA ligands. Among the established NIPP1 FHA ligands, the histone methyltransferase EZH1/2 and the splicing factor SAP155 are good candidates to mediate the testicular phenotype in the iKOs because these proteins have established functions in spermatogenesis^52–54^. However, we cannot exclude that other, hitherto unknown FHA ligands, contribute to the testicular phenotype. At first glance, the notion that the phenotype of *Ppp1r8* ^*−/−*^ testis is due to misregulated dephosphorylation of a subset of PP1 substrates seems at variance with observations that the genetic deletion of PP1α has no overt phenotype^55^. Likewise, the deletion of PP1γ causes male infertility^56,57^, but not a global loss of germ cells as seen after the deletion of NIPP1 (this work). These discrepancies can be explained by functional redundancy between the four PP1 isoforms (PP1α,PP1β, PP1γ1, PP1γ2) and the ability of NIPP1 to form complexes with all PP1 isoforms^58^. This implies that the loss of one PP1 isoform does not preclude the formation of the PP1-NIPP1 holoenzyme and, therefore, does not cause a similarly strong phenotype as the deletion of NIPP1. The male infertility associated with the deletion of PP1γ is not necessarily due to a deficient function of PP1-NIPP1 but is probably caused by the loss of unique holoenzyme complexes between the splice variant PP1γ2 and PP1γ2-specific regulatory proteins in late-meiotic (pachytene spermatocytes) and post-meiotic germ cells^55–57^.

In conclusion, we have shown here that the postnatal deletion of NIPP1 in testis reduces the proliferation and survival potential of spermatogonia and culminates within a few months in the loss of all germ cells. Our data identify NIPP1 as an essential regulator of mammalian spermatogenesis.

## Materials and Methods

### Handling of mice

Mice were housed in a specific-pathogen free facility under standard 12 h light/dark cycles with water and food *ad libitum*. All experiments were approved by the KU Leuven Ethical Committee (P036/2013) and executed according to their guide of care of experimental animals. *Ubc-Cre-ERT2/Ppp1r8* ^*fl/+*^ (CTR) and *Ubc-Cre-ERT2/Ppp1r8* ^*fl/−*^ (iKO) mice were generated using the breeding scheme shown in Supplementary Fig. 1a, involving previously described *Ppp*1r8^+/−^^23^, *Ppp1r8* ^fl/fl^ ^22^ and *Ubc*-*Cre*-*ERT2* mice^24^ (a kind gift from Dr. M. Baes, KU Leuven, Belgium). A germ cell-specific NIPP1 mouse KO model was generated as detailed in Supplementary Fig. 5g, using transgenic *Vasa*-*Cre* mouse^59^ (a kind gift from Dr. D.H. Castrillon, University of Texas Southwestern Medical Center, USA). For genotyping, tail-clip or ear-clip DNA was amplified by PCR, using the primer sets described in Supplementary Table 2. The PCR conditions are available on request.

Tamoxifen (Sigma-Aldrich) was dissolved in 10% (v/v) ethanol in oil at a concentration of 20 mg/ml. Deletion of the floxed *Ppp1r8* allele in adult males was induced by 4 consecutive intraperitoneal injections (every 2 days starting at the age of 4 weeks) of 0.2 mg tamoxifen/g body weight (Fig. 1b). Deletion of the floxed *Ppp1r8* allele in neonates was induced by 3 daily subcutaneous injections, of 0.2 mg tamoxifen/g body weight, starting from 1 day post-partum (dpp), (Supplementary Fig. 7a). BrdU (Sigma) was dissolved in phosphate-buffered saline (PBS) and injected intraperitoneally at 100 mg per kg mouse body weight. Testes were harvested 2 h following BrdU injections. The testes from anaesthetized animals were either directly frozen in liquid nitrogen, fixed in Bouin’s (Sigma-Aldrich) or 4% Paraformaldehyde (PFA) solutions.

### Organ and cell culture

Testis organ culture was performed as described^60,61^. Briefly, testis from adult male mice were decapsulated by removal of the tunica albuginea and subsequently divided with forceps into 4 pieces of approximately 2 mm diameter. The 4 pieces from each testis were placed in one hexahedron of agarose gel and soaked in the organ culture medium containing 2x alpha-MEM (Sigma), 10% knockout serum replacement KSR (Sigma), 100 U/ml penicillin, 100 µg/ml streptomycin and 7% w/v sodium bicarbonate. Testis pieces were maintained in culture for 4 days and 10 µM BrdU (Sigma) was added to the medium 6 hours before harvesting. *In vitro* deletion of the floxed *Ppp1r8* allele was obtained by addition of 2 µM (Z)-4-Hydroxytamoxifen (OHT) (Sigma-Aldrich) to the medium for 96 h. Finally, the 4 pieces of one agarose hexahedron were collectively embedded in paraffin and all 4 pieces were included for the quantification of this experimental condition.

GFRA1-enriched cells (undifferentiated spermatogonia) were isolated from 12 days-old CTR and iKO mice, as described by Guan *et al*. (2009)^41^. Briefly, testicular cells were isolated by an enzymatic procedure using collagenase IV (Worthinghton biochem corp.) and dispase (Invitrogen), followed by enrichment of GFRA1-enriched spermatogonia through laminin (Sigma) selection. This laminin selection method is based on the expression of specific surface markers on SSCs, such as α6 integrin (*Itga6*) and β1-integrins (*Itgb1*). Since some of these receptors bind the extracellular matrix molecule laminin, laminin can be used to enrich testicular cells for SSCs, as reported^41–43^. After enrichment, cells were co-cultured with freshly prepared MEF (mouse embryonic fibroblasts) as feeding cells. The MEFs had been pre-incubated with Mitomycin C (Sigma) and maintained in spermatogonia stem cells (SSCs) growth medium, containing MEM alfa medium (Thermo fisher scientific) and supplemented with 10% FBS, 100 U/ml penicillin, non-essential amino acids (Thermo fisher scientific), β-Mercaptoethanol (Sigma), N2-1 supplement (Thermo fisher scientific), recombinant GDNF (R&D systems; 4 ng/ml) and recombinant LIF (Sigma; 10^3^ U/ml). NIPP1 deletion was induced by addition of 1 µM 4-OHT to the medium for 72 h. For the proliferation assay, 15 µM BrdU was added to the medium 5 hours before harvesting cells. The purity of the cells after laminin enrichment was assessed by quantifying the relative number of GFRA1+ cells in images from GFRA1 and DAPI stained cells. Two independent isolations after laminin selection were scored by selecting 8 images randomly and scoring >180 cells for each isolation.

### Immunohistochemistry

Testes fixed in Bouin’s (6 h) or 4% PFA (24 h) were embedded in paraffin and sectioned at a thickness of 6 µm. Testis sections were stained with Hematoxylin and Eosine (H&E) or the fibrosis marker Sirius red according to standard protocols. DNA was visualized by DAPI or Propidium Iodide (PI), as indicated. For the clarity of the pictures, the blue color of DAPI was often converted to red by image processing. TUNEL assays were performed using the *In-Situ*-Cell-Death-Detection-Kit and Fluorescein (Roche) on paraffin-embedded testis tissue following the instructions of the manufacturer. Testis sections were immunostained using the antibodies listed in Supplementary Table 3 and using the TSA^TM^ Fluorescein, TSA^TM^ Biotin system (PerkinElmer) or chromogenic detection using DAB (3,3′-diaminobenzidine). Detailed immunostaining protocols are available on request. The immunofluorescence images were acquired with a Leica TCS SPE laser scanning confocal system mounted on a Leica DMI 4000B microscope and equipped with a Leica ACS APO 20X objective. Quantifications shown in, Fig. 3b,g and h, Supplementary Fig. 3d and Supplementary Fig. 4b,c were performed by counting the number of cells stained with the indicated cell markers per seminiferous tubule (30 randomly selected tubules per mouse, n = 4). Leica MM AF 2.1 software was used for quantification (10 pictures of 10x objective magnification were randomly selected per mouse testis) of the relative expression of PCNA (Figs 3c and 4f), NIPP1 (Fig. 4b and e, and Supplementary Fig. 1f), SOX9 (Supplementary Fig. 6b), and p16 (Supplementary Fig. 4d) over DAPI (nucleus) signal. Quantification for incorporated BrdU and apoptopic cells in the GFRA1-enriched cell populations (Fig. 6g and h) were performed by counting the number of positive cells relative to the total number of cells in the colony (n = 3; eight colonies for each condition were analysed).

### Biochemical procedures

Testes were homogenized using a dounce homogenizer (Sigma) and incubated in lysis buffer for 20 min at 4 °C. The lysis buffer comprised modified RIPA buffer (50 mM Tris-HCl at pH 7.4, 1% Triton-X 100, 0.2% sodium deoxycholate, 0.2% sodium dodecyl sulfate (SDS), 1 mM EDTA, 0.3 M NaCl) or SDS-lysis buffer (50 mM Tris-HCl at pH 7.4, 2% SDS, 12% glycerol, 100 mM β-mercaptoethanol). Lysis buffers were supplemented with 20 mM NaF, 5 µM leupeptin, 0.5 mM phenylmethanesulfonyl fluoride, 0.5 mM benzamidine and 1 mM orthovanadate. The lysates were centrifuged for 5 min at 1800 × *g* and the supernatant was used for immunoblotting. The testis extracts for immunoblotting in Figs 1e, 2c, 3d and Supplementary Fig. 5 were prepared with modified RIPA, while SDS-lysis buffer was used in Supplementary Fig. 7c. Immunoblotting was performed following 10% SDS- PAGE with the indicated antibodies (Supplementary Table 3). Immunoblots were visualized using ECL reagent (Perkin Elmer) in an ImageQuant LAS4000 imaging system (GE Healthcare) (see Supplementary Fig. 8 for uncropped blots) and were quantified using ImageQuant TL software (GE Healthcare).

### RNA sequencing and Quantitative Reverse Transcriptase PCR (qRT-PCR)

Total RNA was isolated from 40 mg of snap-frozen mouse testis using the GenElute^TM^. Mammalian Total RNA Miniprep kit (Sigma-aldrich). RNA integrity of the samples used for RNA sequencing was assessed using a Bioanalyser 2100 (Agilent). Library preparation, sequencing and statistical analysis of the RNA sequencing data were performed by VIB Nucleomics Core, as detailed in the Supplementary information.

Complementary DNA (cDNA) was synthetized from 2 µg of total RNA using oligo dT primers (Sigma-aldrich) RevertAid Premium Reverse Transcriptase and RiboLock RNase inhibitor enzymes (Fermentas). About 1.2% of the cDNA was amplified by PCR in duplicate using SYBR Green qPCR Mix (Invitrogen) and a Rotorgene detection system (Corbett Research). To compare the relative amount of target genes in different samples, values were normalized to the housekeeping gene *Hprt* (Hypoxanthine-guanine phosphoribosyltransferase) or a cell-type specific gene as indicated in the Figures. qRT-PCR primers are listed in Supplementary Table 4.

### Statistical analysis

All statistical analysis was performed using GraphPad Prism software. Two-way unpaired (Figs 1–5 and 6i–l, Supplementary Figs 1–3 and 4–6) or paired (Fig. 6e–h) student’s t-test, and Pearson’s correlation test (Supplementary Fig. 6f) were used.

### Data availability

All gene expression data are available at GEO under the accession number GSE83145.

## Electronic supplementary material

Supplementary information

Database 1
