# Supplementary material for: The protein phosphatase 1 regulator NIPP1 is essential for mammalian spermatogenesis
Source: Sci Rep. 2017 Oct 17;7:13364. doi: 10.1038/s41598-017-13809-y (PMC5645368; doi:10.1038/s41598-017-13809-y)
Supplement: Supplementary file 1 — Supplementary information [file 41598_2017_13809_MOESM1_ESM.pdf]

The protein phosphatase 1 regulator NIPP1 is essential for mammalian spermatogenesis

Mónica Ferreira[1,2], Shannah Boens[1], Claudia Winkler[1], Kathelijne Szekér[1], Iris Verbinnen[1], Aleyde Van Eynde[1,*], Margarida Fardilha[2] and Mathieu Bollen[1,*]

[1]Laboratory of Biosignaling & Therapeutics, KU Leuven Department of Cellular and Molecular Medicine, University of Leuven, Leuven, Belgium.
[2]Institute for Research in Biomedicine-iBiMED, Health Sciences Department, University of Aveiro, Aveiro, Portugal.

*Correspondence: Mathieu.Bollen@kuleuven.be; Aleyde.VanEynde@kuleuven.be

Supplementary information

Supplementary experimental procedures

Testosterone assay

Serum testosterone was determined at the Centre for Laboratory Medicine (Laboratoriumgeneeskunde UZ-KU Leuven, Belgium). Blood was extracted by cardiac puncture from male mice of 9 weeks that had been housed with an adult female for 2 weeks.

RNA sequencing

The Nanodrop ND-1000 (Nanodrop Technologies) was used to determine the RNA concentration and purity. RNA integrity was examined with the Bioanalyser 2100 (Agilent). Library preparation, sequencing and analysis were performed by the VIB Nucleomics Core (www. nucleomics.be) according to their standard protocols. Briefly, 5 µg of total RNA per sample was used as input. rRNA was depleted using Illumina TruSeq® Stranded Total RNA Sample Prep Kit with Ribo-Gold (Illumina). cDNA was generated using random primers and double stranded cDNA was synthesized using DNA polymerase I and RNAase H. Next, multiple indexing adapters were ligated to introduce different barcodes for each sample, followed by an enrichment PCR. The sequence libraries of each sample were equimolarly pooled and sequenced (1/2 run of iIllumina NextSeq500 flow-cell at 2x 75 bp). Preprocessing was performed using FastX 0.013, Cutadapt 1.7.1 and Short read 1.16.3 to remove mainly low quality ends, adaptor sequences and unreliable reads [1–4]. For the mapping of the reads alignment with Tophat v2.0.13 to the reference genome of *Mus musculus* (GRCm38.73) was performed [5]. Reads with a mapping quality smaller than 20 were removed from the alignments using Samtools 1.1 [6]. Transcript coordinates were extracted from the GRC reference annotation (Gffread from the Cufflinks v2.1.1 suite), and merged to gene coordinates (mergeBed from the Bedtools v2.17.0 toolkit). Next, the number of aligned reads per gene was counted (HTSeq-count v0.6.1p1) [7,8]. Genes for which all samples had less than 1 cpm (count-per-million) were removed. Raw counts for the retained 17.344 genes were further corrected within samples for GC-content and between samples using full quantile-normalization, according to the EDASeq package from Bioconductor [9]. Differential gene expression was determined with the EdgeR 3.4.0 package by fitting a negative binomial generalized linear model (GLM) against the normalized counts [10]. Differential expression was tested for with a GLM likelihood ratio

test and the resulting p-values were corrected for multiple testing with Benjamini-Hochberg to control the false discovery rate [11].

Supplementary Figures

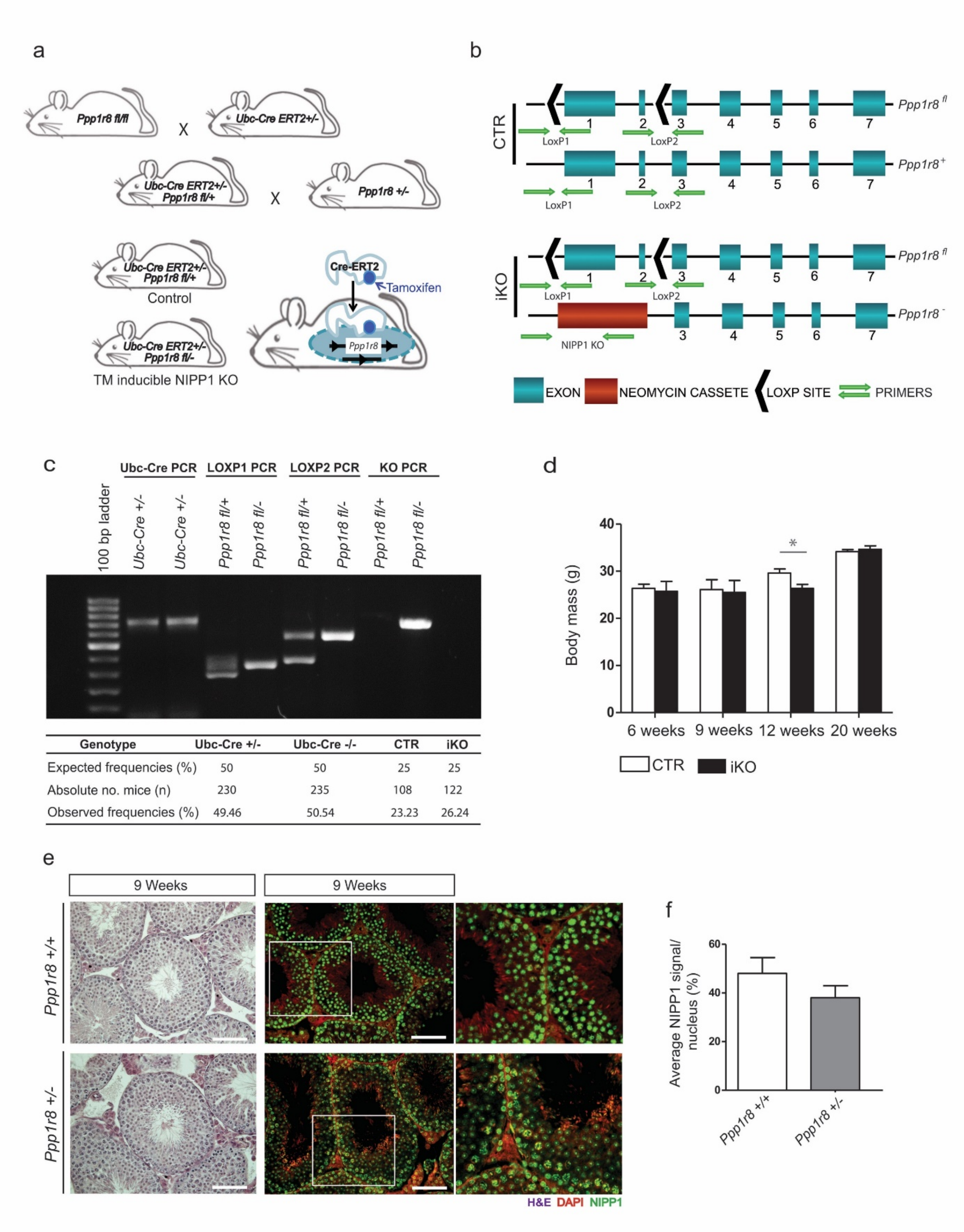

Supplementary Figure 1. Breeding strategy and mice genotyping for inducible deletion of NIPP1. (a) Scheme of crossing strategy for the generation of the inducible *Ppp1r8* knockout mice (iKO) and their controls (CTR). The administration of tamoxifen

causes the nuclear translocation of CRE-ERT2 [12]. **(b)** Scheme of the localization of the primers used for PCR-based genotyping of the CTR and iKO. **(c)** Representative example of PCRs on genomic tail DNA used for genotyping CTR and iKO. The primer sequences are shown in Supplemental Table 2. Also indicated are frequencies of the offspring from the *Ubc-Cre-ERT2*$^{+/-}$ *Ppp1r8*$^{fl/+}$ and *Ppp1r8*$^{+/-}$ mice crosses. **(d)** Body mass of tamoxifen-injected CTR and iKO mice at 6, 9, 12 and 20 weeks. *p<0.05. **(e)** Hematoxylin-Eosin staining (left panels), staining for DNA (DAPI, red) and immunostainings of NIPP1 (right panels) in testis from wild-type (WT) mice and *Ppp1r8*$^{+/-}$ mice at 9 weeks. The right pictures are higher magnification of the squares in the middle pictures. Bar, 50 µm. **(f)** Quantification of NIPP1 immunostainings as illustrated in panel (e). The data represent means ± SEM (n=4).

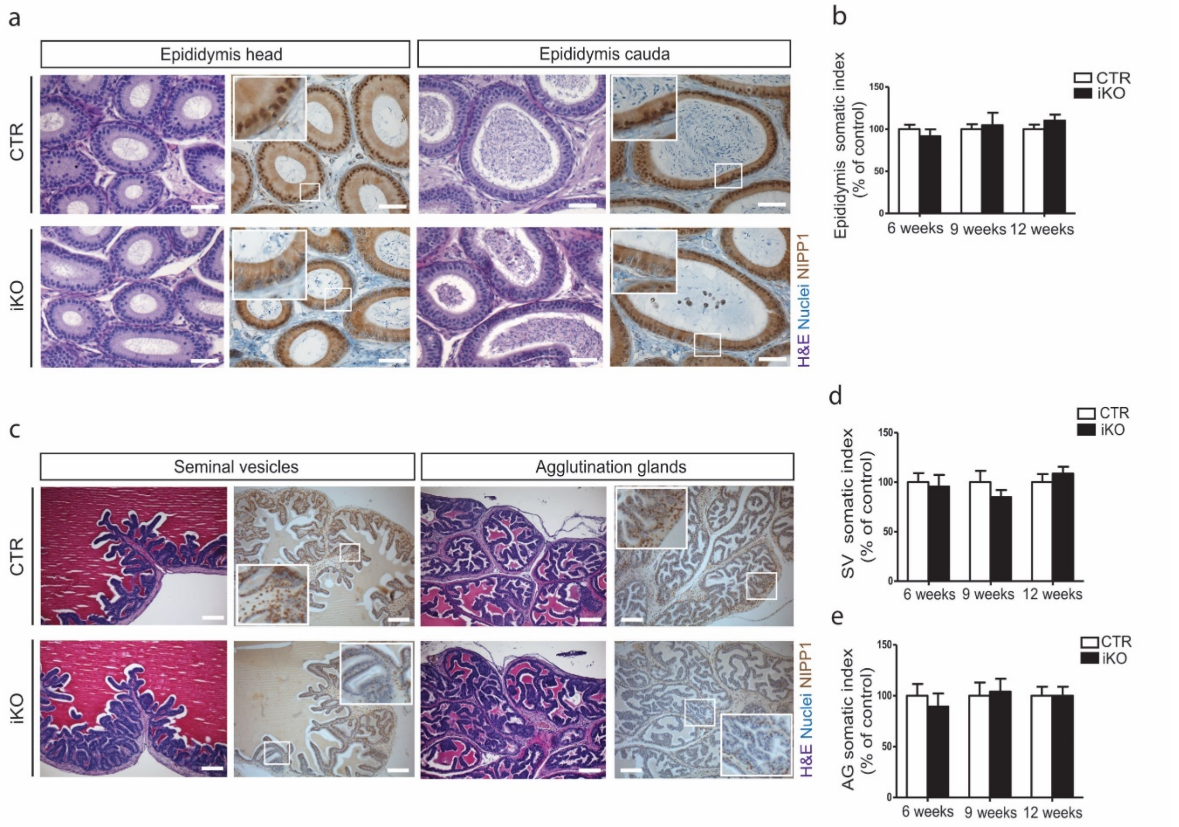

Supplementary Figure 2. The somatic index of the epididymis and the male accessory glands are not affected by the tamoxifen-induced deletion of NIPP1. **(a)** Sections of the epididymis head and cauda from tamoxifen-injected mice at the age of 9 weeks were stained for Hematoxylin-Eosin (H&E) and immunostained for NIPP1 (chromogenic DAB detection), with a Hematoxylin counterstain for the visualization of nuclei. Scale bars represent 50 µm in all panels of this figure. The insets (big squares) show enlargements of the small squares. **(b)** The epididymis somatic index of tamoxifen-injected CTRs and iKOs at the indicated time points. The somatic index was determined by the percentage of total organ weight (g) over the body weight (g), and was expressed as a % of the value for the CTRs. All data are represented as means ± SEM (n=4). **(c)** Sections of seminal vesicles (SV) and agglutination glands (AG) from tamoxifen-injected CTR and iKO mice at the age of 9 weeks. The sections were stained with H&E and immunostained for NIPP1 (chromogenic DAB detection) with a Hematoxylin counterstain for the visualization of nuclei. **(d)** The SV somatic index of tamoxifen-injected CTRs and iKOs at the indicated time points. **(e)** The AG somatic index of tamoxifen-injected CTRs and iKOs at the indicated time points.

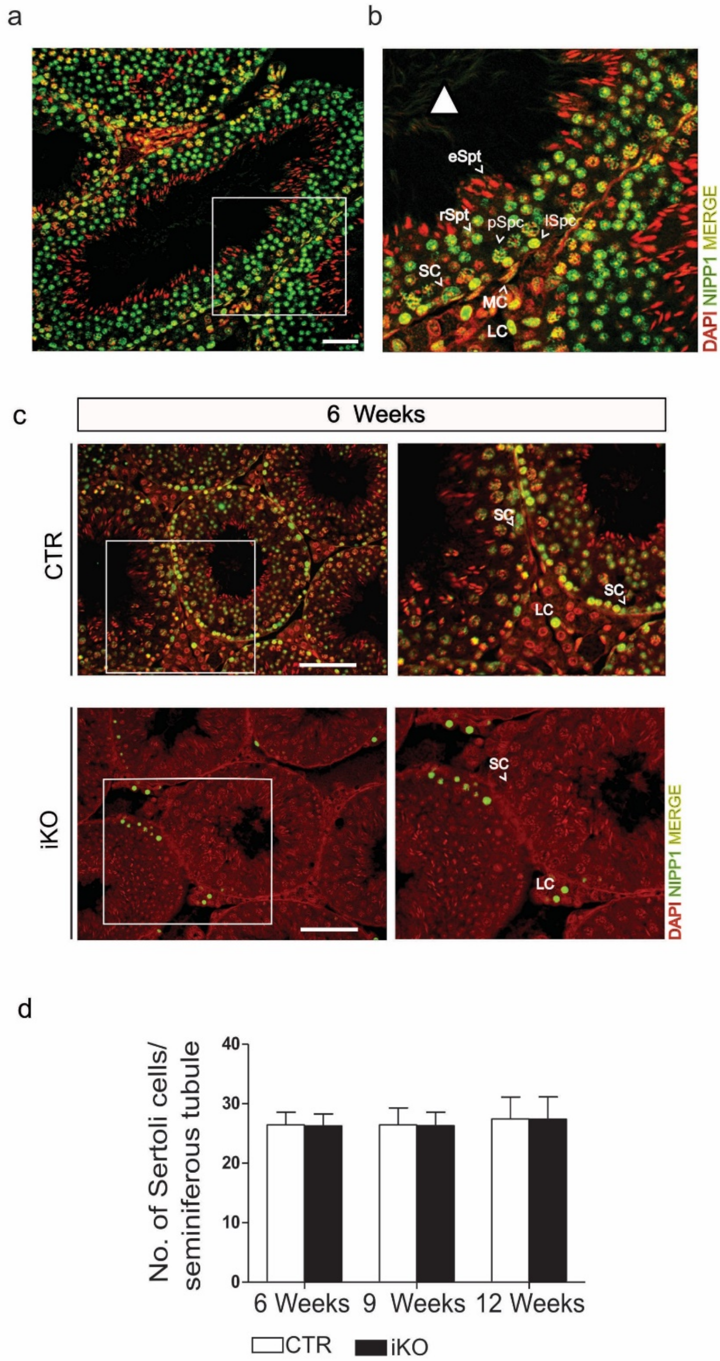

Supplementary Figure 3. Expression of NIPP1 in testis of adult mice. (a) Immunostainings of NIPP1 in testis from wild-type mice at the age of 6 weeks. Scale bar, 25 µm. **(b)** Higher amplification of the square from panel (a). LC, Leydig cells; MC, Myoid cells; SC, Sertoli cells; Spc, spermatocytes; pSpg, pachetene spermatogocytes; lSpc, leptotene spermatocytes; eSpt, elongated spermatids; rSpt, round spermatids. White triangle, lumen. **(c)** Magnifications of CTR and iKO testis sections of the same pictures as shown in the left panel of Figure 1c. The sections from 6 weeks old mice that were tamoxifen injected at 4 weeks were immunostained for NIPP1, demonstrating

that NIPP1 is expressed in germ cells and somatic cells of the seminiferous tubules and interstitial cells in the CTR mice and is efficiently deleted in the iKO mice. In all panels of this figure, DAPI was used to visualize nuclei. SC, Sertoli cells; LC, Leydig cells. **(d)** The number (No.) of Sertoli cells per seminiferous tubule.

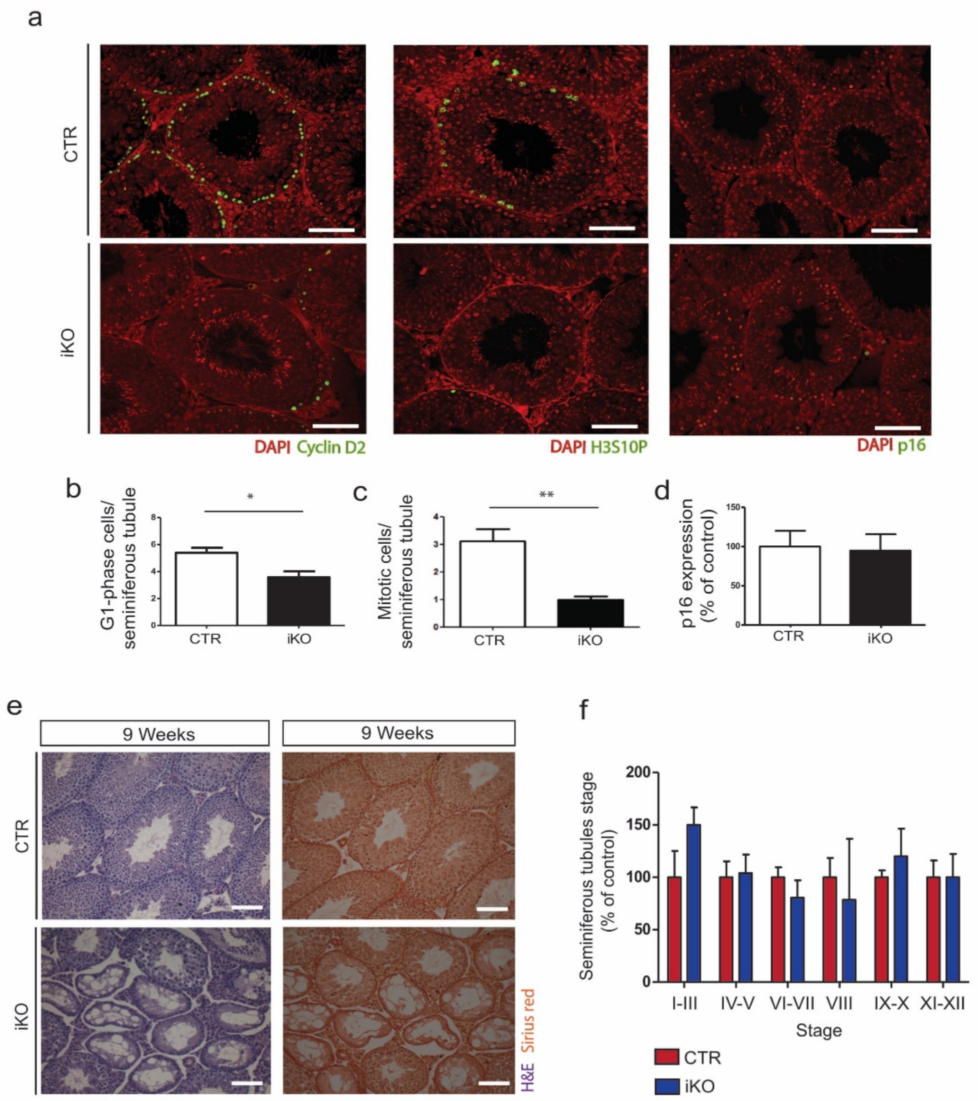

Supplementary Figure 4. The testicular loss of NIPP1 results in hypoproliferation but not senescence or fibrosis. (a) Testis sections of tamoxifen-treated mice of 6 weeks were stained for DNA (DAPI), Cyclin D2, phospho-Ser 10 of Histone H3 (H3S10P) and p16. Scale bar, 50 µm. **(b)** Quantification of G1-phase cells by counting the number of Cyclin D2 positive cells per seminiferous tubule of immunostainings as shown in panel (a). **(c)** Quantification of mitotic cells by counting the number of H3S10P positive cells of immunostaining as shown in panel (a). **(d)** Quantification of immunostainings of the senescence marker p16, as shown in panel (a). All data are represented as means ± SEM (n=4). *, *p*<0.05; **, *p*<0.01. **(e)** Testis sections of tamoxifen-treated mice of 6 weeks were H&E stained and stained for fibrosis with Sirius red. Scale bar, 50 µm. **(f)** Quantification of the indicated seminiferous tubule stages (I-XII) in testis sections of tamoxifen-treated mice at the age of 6 weeks [13].

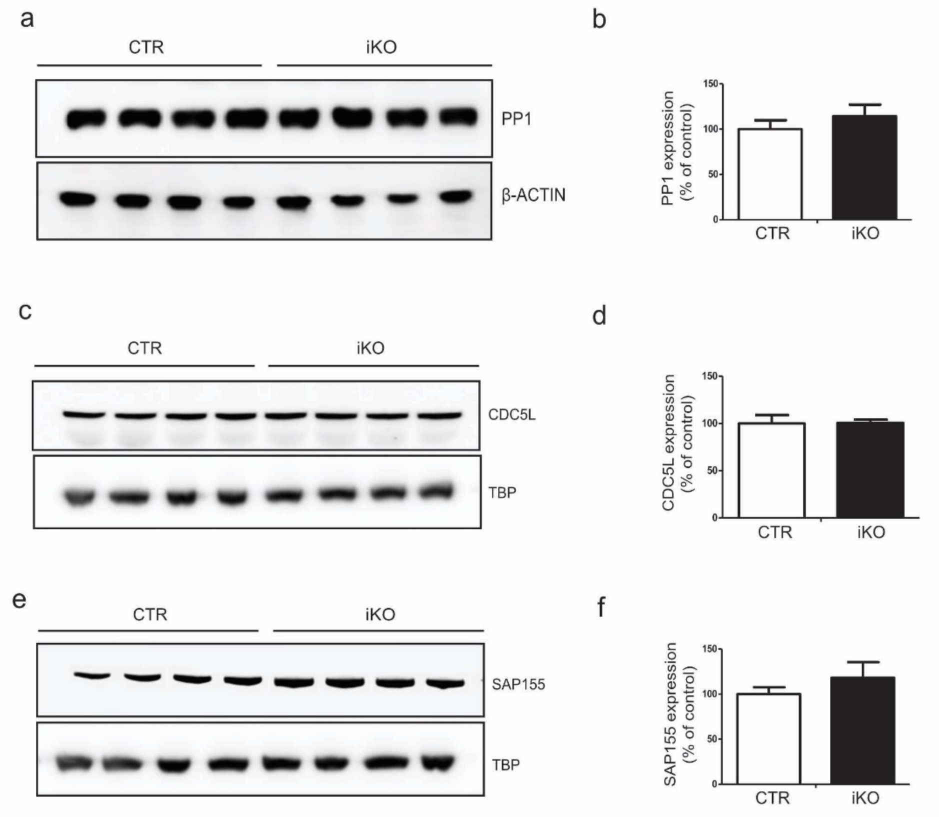

Supplementary Figure 5. The level of the NIPP1 interactors PP1, CDC5L and SAP155 is not altered by the tamoxifen-induced deletion of NIPP1. PP1 **(a,b)**, CDC5L **(c,d)** and SAP155 **(e,f)** in total testis extracts from tamoxifen-injected CTR and iKO mice at the age of 6 of weeks was visualized by immunoblotting (left panels) and quantified (right panels). The PP1 antibody recognizes all three isoforms of PP1 (α, β, γ). β-actin and TBP were used as a loading control.

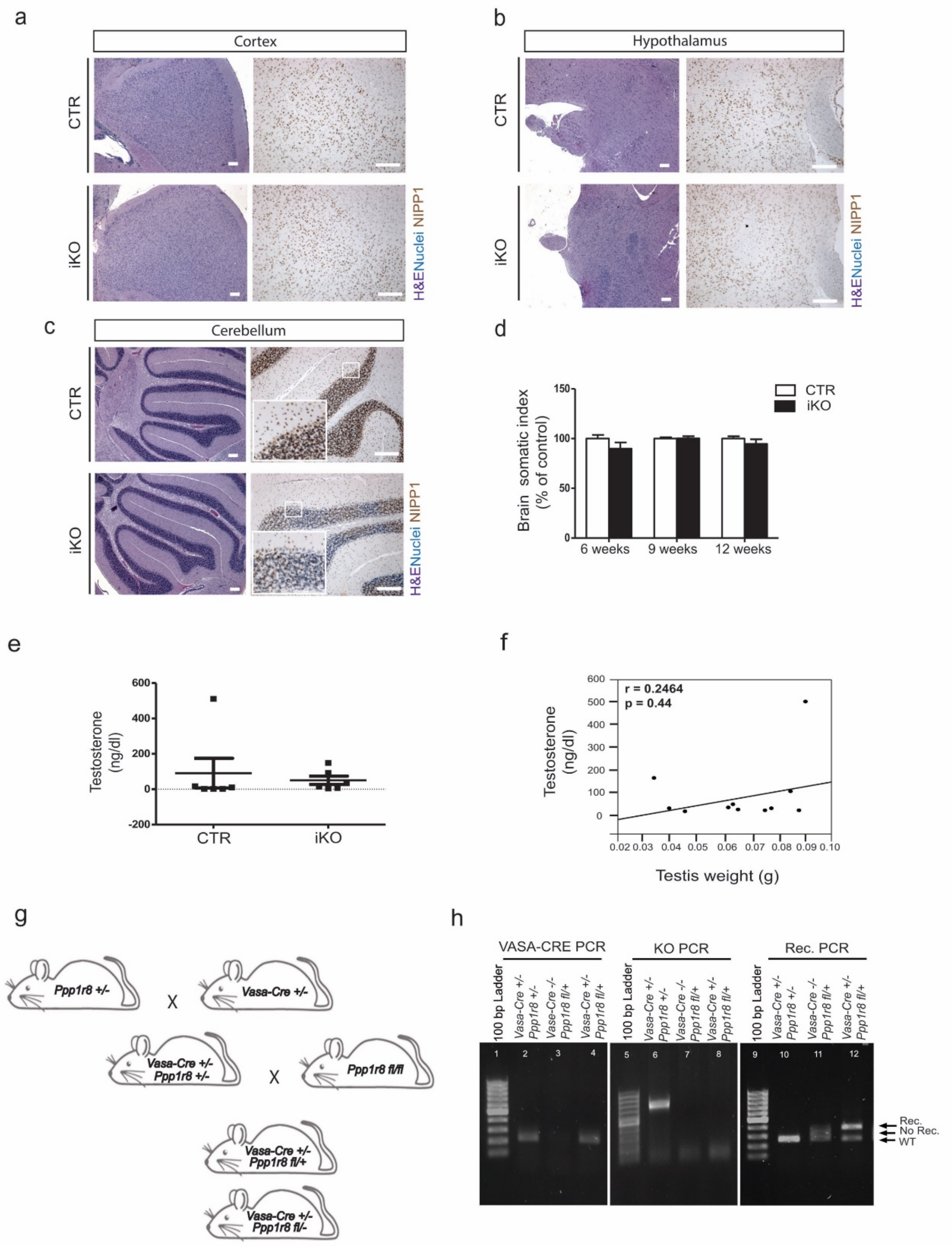

Supplementary Figure 6. The testis phenotype is an intrinsic testicular defect. **(a)** Brain cortex sections of tamoxifen-treated mice of 6 weeks were Hematoxylin-Eosin (H&E) stained and immunostained for NIPP1 (chromogenic DAB detection), with a Hematoxylin counterstain for the visualization of nuclei. Scale bars represent 100 µm in all panels of this Figure. **(b)** Hypothalamus sections of tamoxifen-treated mice of 6 weeks were stained for H&E and NIPP1 DAB staining. **(c)** Cerebellum sections of tamoxifen-

treated mice of 6 weeks were stained for H&E and NIPP1 DAB staining. **(d)** The brain somatic index of tamoxifen-treated CTR and iKO mice at the indicated timepoints. **(e)** Testosterone levels in blood serum of tamoxifen-treated mice of 6 weeks. Data are means ± SEM (n=6). **(f)** Scatter plot of the correlation between testis weight (g) and blood testosterone basal levels (ng/dl). Pearson's correlation test: *r*, correlation coefficient; P, *p* value. **(g)** Scheme of breeding strategy for the generation of the testis specific *Ppp1r8* knockout mice using the transgenic *Vasa-Cre* mice. **(h)** Representative example of the PCR amplicons derived from PCR on tail DNA from mice with the indicated genotypes. The following primers were used: primers *VasaCre* forward and *VasaCre* reverse in lanes 2-4; primers NIPP1 KO forward and NIPP1 KO reverse in lanes 6-8 and primers LoxP1 forward, LoxP1 reverse, and LoxP2 reverse in lanes 10-12. The efficiency and specificity of *Vasa-Cre*-recombinase is shown in lanes 10-12, where the resulting PCRs amplicons which correspond to *Ppp1r8* wild-type (WT; 267 bp), no recombined *Ppp1r*8^{fl} (No Rec; 329 bp) and recombined *Ppp1r8*^{fl} (Rec; 389 bp) are represented. This indicates that recombination occurred in the tail of VasaCre$^{+/-}$ /*Ppp1r8*$^{fl/-}$ mice, due to precocious activity of VasaCre recombinase, implying that this knockout model is not germ-cell specific. The primer-sequence and location of the primers are indicated in Supplemental Fig. 1 and Supplementary Table 2. bp, base pairs; fl, loxP allele.

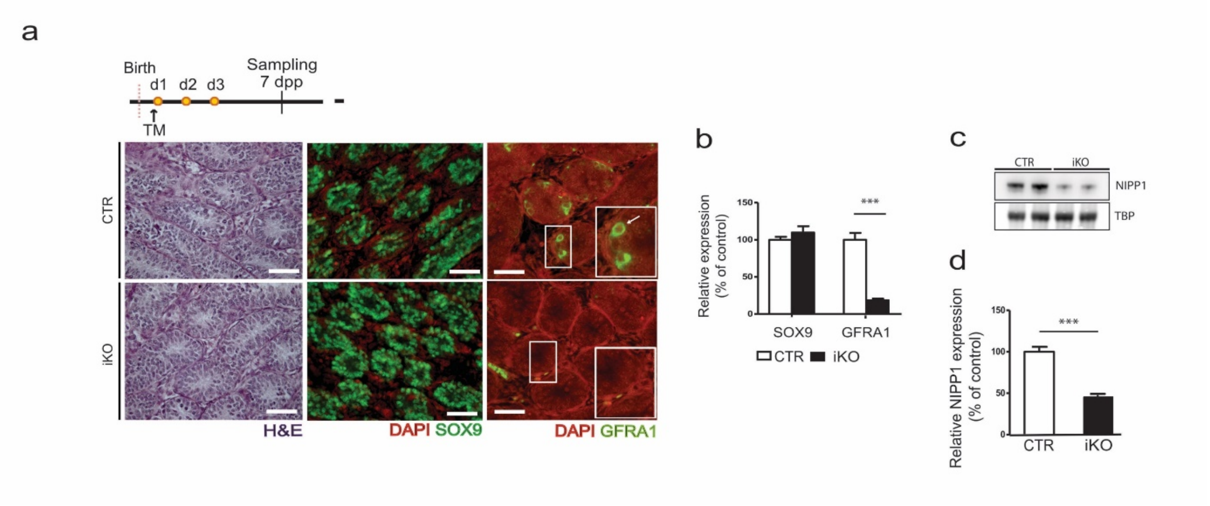

Supplementary Figure 7. The removal of NIPP1 from neonatal testis leads to the loss of undifferentiated spermatogonia. (a) Scheme of tamoxifen induction and sampling of testis from neonates. UBC-CRE-ERT2 driven deletion of *Ppp1r8* was induced by 3 consecutive daily subcutaneous injections of 0.2 mg tamoxifen/g mouse starting at 1 day post-partum (dpp). TM, tamoxifen. Testis sections from neonates of 7 days that had been treated with tamoxifen were stained for H&E, DAPI, SOX9 and GFRA1. The insets (big squares) show enlargements of the small squares. Scale bar, 50 µm. **(b)** Quantifications of stainings as shown in panel (a) (n=4). **(c)** Testis lysates from the same mice were used for immunoblotting with the indicated antibodies. TBP was used as a loading control. **(d)** Quantifications of NIPP1 expression as shown in panel (c) (n=4). ***, p<0.001. Full-length blots are presented in Supplementary Fig. 7.

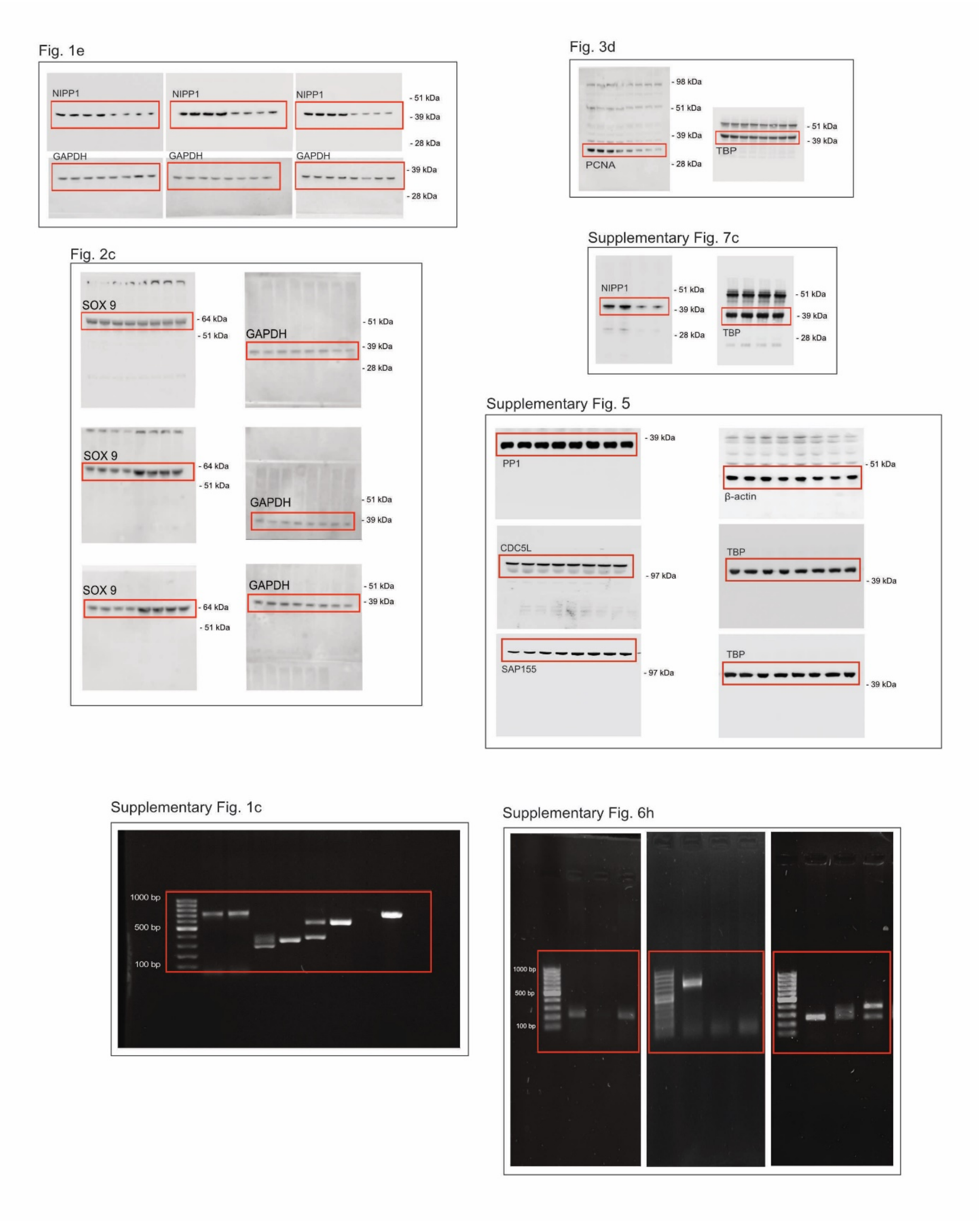

Supplementary Figure 8. Uncropped images of immunoblots as acquired with the ImageQuant LAS4000 imaging system (GE Healthcare). Red boxes show approximate image used for presentation.

Supplementary Tables

Supplementary Table 1. Differentially expressed genes in iKO mice of 6 weeks, as derived from the RNA sequencing data. Genes are arranged by descendent order of FDR value.

UPREGULATED GENES						DOWNREGULATED GENES					
GENE ID	FC	FDR	GENE ID	FC	FDR	GENE ID	FC	FDR	GENE ID	FC	FDR
Sgsh	3.814	4.344E-18	Phkg1	3.296	1.177E-02	Cuzd1	0.1858	6.6E-27	Try5	0.4637	5.1E-03
Adh1	2.150	5.919E-14	Wfs1	2.731	1.217E-02	Slc9c1	0.5227	2.0E-25	Cenpt	0.5038	5.1E-03
C1qtnf4	1.611	2.010E-12	*Adamtsl1*	1.547	1.224E-02	*Gmcl1*	0.5875	2.0E-20	*Gipc2*	0.4858	5.2E-03
Rgs9	3.396	2.942E-10	*Arhgap30*	1.545	1.263E-02	*Tdrd9*	0.6161	4.3E-18	*Nlrp4c*	0.4366	5.3E-03
Ccnb3	2.184	1.857E-09	*Nlrp10*	4.526	1.424E-02	*Lrpprc*	0.5435	7.5E-18	*Dnph1*	0.2875	5.3E-03
Nmnat3	1.592	1.708E-07	*Lrsam1*	1.559	1.501E-02	*Gm13941*	0.6049	2.8E-17	*Pigv*	0.6283	5.4E-03
Ttn	1.559	2.245E-06	*Otud6a*	1.509	1.547E-02	*Cpsf1*	0.6317	1.7E-13	*A1cf*	0.5035	6.0E-03
Leng8	1.565	2.356E-06	*Ptprt*	1.533	1.586E-02	*Hist1h1t*	0.5530	7.4E-12	*Hist1h2ak*	0.4407	6.1E-03
Ppp1r1c	1.617	2.501E-06	*Npr2*	1.855	1.615E-02	*Kntc1*	0.5816	7.5E-12	*Myh13*	0.3547	6.7E-03
Zfp940	1.955	4.534E-06	*Slc1a4*	2.774	1.624E-02	*Tuba3a*	0.6482	3.4E-10	*Pop5*	0.5532	6.7E-03
Prss35	1.716	7.238E-06	*Nfatc2*	1.862	2.087E-02	*Wdr63*	0.6622	1.6E-09	*Mal*	0.5788	8.0E-03
Pmp2	2.266	9.900E-06	*Bbc3*	2.758	2.172E-02	*Abca2*	0.3703	2.7E-09	*Large*	0.6120	9.8E-03
Prdm8	2.630	1.032E-05	*Abcg8*	2.100	2.260E-02	*Zbtb17*	0.6102	4.0E-09	*Adam7*	0.0867	1.1E-02
Nsun5	1.931	1.131E-05	*Mypn*	1.588	2.465E-02	*Ces5a*	0.1387	6.4E-09	*Jph1*	0.3192	1.1E-02
Trpv1	2.573	1.962E-05	*Icam2*	3.094	2.479E-02	*Wdr11*	0.6369	6.6E-09	*Rundc3b*	0.5938	1.1E-02
Slc1a3	1.801	3.031E-05	*Agmo*	2.574	2.527E-02	*Daw1*	0.6004	7.4E-09	*Cdkn2a*	0.3445	1.1E-02
Pet2	1.788	3.566E-05	*Xrcc3*	1.729	2.527E-02	*Ercc2*	0.6209	1.7E-08	*Hist1h2bj*	0.3960	1.1E-02
Dact1	1.979	3.605E-05	*Dtx3l*	1.650	2.664E-02	*Dnhd1*	0.6257	2.3E-08	*Prtg*	0.2723	1.1E-02
Hist2h2be	1.566	3.615E-05	*Hsd3b6*	1.947	2.740E-02	*Sord*	0.4173	4.6E-08	*Prpsap2*	0.6400	1.2E-02
Ndufs5	8.252	5.923E-05	*Atn1*	2.221	2.794E-02	*Suox*	0.4370	1.2E-07	*Kctd4*	0.3809	1.3E-02
Farp1	2.226	8.815E-05	*Mmp15*	2.186	2.884E-02	*Hspa12a*	0.5700	1.4E-07	*Dnd1*	0.4309	1.3E-02
Bdkrb2	6.224	1.513E-04	*Unc79*	1.761	2.941E-02	*Spink8*	0.0417	1.4E-07	*Trim71*	0.5301	1.4E-02
Slc35c1	2.907	1.720E-04	*Hcn1*	3.993	2.983E-02	*Cd52*	0.1227	1.7E-07	*Hist2h3b*	0.2816	1.4E-02
Agr3	9.795	2.367E-04	*Sema4g*	1.877	2.983E-02	*Crisp1*	0.0396	2.7E-07	*Wfdc13*	0.0965	1.4E-02
Fam115a	1.552	2.397E-04	*Csmd2*	1.550	3.014E-02	*Hist1h2ba*	0.6530	5.1E-07	*Wfdc15b*	0.1410	1.4E-02
Ltbr	1.511	2.445E-04	*Deaf1*	1.565	3.075E-02	*Glb1l2*	0.3709	6.5E-07	*Lin28b*	0.6467	1.4E-02
Pmfbp1	1.600	2.700E-04	*Krt10*	1.681	3.089E-02	*Haghl*	0.6367	9.4E-07	*Grik3*	0.3156	1.5E-02
Ipo5	1.787	2.728E-04	*Car3*	3.403	3.179E-02	*Pnkp*	0.5810	1.9E-06	*Hist1h2bk*	0.4319	1.5E-02
Sult1e1	2.590	3.008E-04	*Cml1*	2.105	3.262E-02	*Prss56*	0.5915	2.3E-06	*Cdc42bpg*	0.6516	1.5E-02
Gfap	1.846	3.140E-04	*Casp1*	2.764	3.527E-02	*Epsti1*	0.6406	4.1E-06	*Gpr82*	0.3505	1.5E-02
Gldn	5.541	3.745E-04	*Cuedc1*	2.108	3.679E-02	*Gclc*	0.6557	5.7E-06	*Kifc5b*	0.5568	1.6E-02
Acp1	2.832	4.015E-04	*Fbln2*	1.811	3.829E-02	*Gm4735*	0.3668	6.7E-06	*Apoa2*	0.2225	1.6E-02
Chgb	1.635	4.518E-04	*Fancb*	3.216	3.892E-02	*Rrm2*	0.6324	1.8E-05	*Sarm1*	0.2913	1.6E-02
Jph2	1.763	4.561E-04	*Lhx9*	1.799	3.939E-02	*Hba-a1*	0.3332	2.1E-05	*Ccdc36*	0.6671	1.7E-02
Ptchd3	1.847	5.655E-04	*Myh14*	1.810	3.973E-02	*Guca2b*	0.1137	3.1E-05	*Park2*	0.6201	1.8E-02
Clec11a	1.910	6.322E-04	*C8a*	3.194	3.978E-02	*Orc2*	0.6620	6.7E-05	*Defb23*	0.0968	1.8E-02
Pdlim7	2.526	6.996E-04	*Anks4b*	1.846	3.978E-02	*Smco2*	0.6298	1.3E-04	*Hbb-b1*	0.5176	1.9E-02
Kitl	1.522	8.880E-04	*Nfic*	1.548	4.068E-02	*Hist3h2ba*	0.2640	1.3E-04	*Cbx2*	0.5446	2.0E-02
Klk1b21	2.018	9.406E-04	*Arhgef15*	1.602	4.129E-02	*Serpina1f*	0.1382	1.6E-04	*Zfp524*	0.6491	2.1E-02
Flot2	1.551	9.491E-04	*Pyroxd2*	1.881	4.143E-02	*Spc25*	0.6449	1.6E-04	*Acad10*	0.6039	2.2E-02
Asb2	1.780	9.541E-04	*Camk1g*	1.694	4.143E-02	*Hist1h2bm*	0.3920	2.0E-04	*Grtp1*	0.1032	2.2E-02
Adam23	1.845	9.798E-04	*Glipr2*	1.714	4.353E-02	*Krt18*	0.1505	2.5E-04	*Spint5*	0.3962	2.4E-02
Agt	1.736	1.058E-03	*Gapt*	3.653	4.458E-02	*Plekhg4*	0.5809	2.7E-04	*Hist1h2ai*	0.4636	2.4E-02
Col17a1	1.979	1.417E-03	*Dsp*	1.503	4.572E-02	*Spint1*	0.2862	4.3E-04	*Plekha4*	0.6682	2.4E-02
Cacng5	5.934	1.940E-03	*Loxl2*	1.691	4.834E-02	*Gucy2g*	0.4166	4.8E-04	*Trpv6*	0.3154	2.4E-02
Gjd3	2.242	1.988E-03				*Cpn1*	0.1024	5.1E-04	*Itpa-ps1*	0.6288	2.4E-02
Artn	1.803	2.096E-03				*Spdyb*	0.6366	5.1E-04	*Grm6*	0.4034	2.5E-02
Parp8	1.647	2.148E-03				*Myh6*	0.3827	6.2E-04	*Dnmt3l*	0.6592	2.7E-02
Adprm	1.544	2.611E-03				*Hist1h4n*	0.4344	7.5E-04	*Nlrp4f*	0.4994	2.8E-02
Gipc3	4.025	2.611E-03				*Thoc6*	0.4894	7.5E-04	*Nanos1*	0.3539	2.9E-02
Mageb3	1.918	2.615E-03				*Ddx43*	0.6380	1.0E-03	*Pcyox1l*	0.4139	2.9E-02
Pomc	1.627	2.615E-03				*Myoc*	0.2787	1.1E-03	*Dhps*	0.6467	3.0E-02
Klk1b27	2.600	2.902E-03				*Cdo1*	0.5676	1.1E-03	*Cx3cr1*	0.6635	3.1E-02
Vat1l	2.216	2.922E-03				*Pold1*	0.4964	1.2E-03	*Grb7*	0.1383	3.1E-02
Rbp7	3.346	2.991E-03				*Hmga1*	0.5587	1.4E-03	*Gpm6a*	0.5665	3.1E-02
Man1a	1.549	3.087E-03				*Pate2*	0.5182	1.4E-03	*Hist1h4j*	0.5316	3.1E-02
Zbtb39	1.609	3.213E-03				*Ros1*	0.5719	1.6E-03	*Cd247*	0.5862	3.2E-02
Wdr91	2.126	3.709E-03				*Uba1y*	0.6377	1.6E-03	*Hbb-b2*	0.3539	3.4E-02
Cd93	1.663	4.431E-03				*Tyk2*	0.6678	1.7E-03	*Abcb5*	0.1381	3.5E-02
Ryr1	1.595	4.544E-03				*Hist1h2aa*	0.6413	1.7E-03	*Sall4*	0.3891	3.6E-02
Eps8l2	2.111	4.713E-03				*Wdhd1*	0.6007	1.9E-03	*Trhde*	0.5816	3.7E-02
Dsc3	1.584	5.123E-03				*Mast3*	0.5393	3.0E-03	*Cdhr2*	0.4974	3.8E-02

Tnk1	2.320	5.138E-03	*Cdca7*	0.4558	3.0E-03	*Tmem25*	0.5274	4.1E-02
Il21	2.612	5.369E-03	*Hmga1-rs1*	0.5868	3.1E-03	*Gprc5c*	0.5132	4.1E-02
Tktl1	1.874	5.394E-03	*Lat2*	0.5559	3.3E-03	*Prom2*	0.1414	4.2E-02
Klk1b24	2.113	5.617E-03	*Defb28*	0.1006	3.5E-03	*Nkx2-4*	0.6329	4.2E-02
P4htm	1.781	6.112E-03	*Dusp9*	0.2509	3.5E-03	*Bbox1*	0.5955	4.3E-02
Wdsub1	1.540	6.112E-03	*Ap4s1*	0.6326	3.5E-03	*Ankrd13d*	0.4343	4.4E-02
Otog	6.190	6.112E-03	*Fancl*	0.6688	3.6E-03	*Elovl7*	0.3663	4.4E-02
Il20rb	2.986	6.658E-03	*Car4*	0.3578	3.7E-03	*Kifc1*	0.6595	4.4E-02
Myo7b	1.851	8.051E-03	*Hist1h2bb*	0.3460	3.9E-03	*Hist1h2ah*	0.3186	4.5E-02
Slc6a15	4.248	8.599E-03	*Polr3gl*	0.6650	4.2E-03	*Mcm2*	0.6028	4.5E-02
Ampd3	1.656	9.019E-03	*Spocd1*	0.5582	4.2E-03	*Wfdc6b*	0.3801	4.6E-02
Cdsn	2.581	9.706E-03	*Hist1h4i*	0.6000	4.3E-03	*Gpr68*	0.4121	4.9E-02
Slc38a6	1.632	9.706E-03	*Rpia*	0.6292	4.7E-03	*Defb22*	0.1461	5.0E-02
Kcnn3	2.462	1.102E-02	*Hist1h1a*	0.5801	4.7E-03			
Kif2b	1.762	1.109E-02	*Pou3f3*	0.15101	4.9E-03			

*FC, fold change; FDR, false discovery rate.

Supplementary Table 2. Primers used for genotyping CTR and NIPP1 KO mice.

*Ubc*Cre forward	GCCTGCATTACCGGTCGATGCAACGA
*Ubc*Cre reverse	GTGGCAGATGGCGCGGCAACACCATT
VasaCre forward	CACGTGCAGCCGTTTAAGCCGCGT
VasaCre reverse	TTCCCATTCTAAACAACACCCTGAA
LoxP1 forward	CTTACAAGGAGTGGTATTCGAACC
LoxP1 reverse	ACTGTCTAGCAGGGCATAGTGTTG
LoxP2 forward	CCACCCTCTCCTTTACTTTGTCTTC
LoxP2 reverse	GGAGAGGAGTAATGAGAGGAGTTGTG
NIPP1 KO forward	CCTCAGCAGATAGCCCACGG
NIPP1 KO reverse	CGCATCGCCTTCTATCGCCTTCTTGAC

Supplementary Table 3. List of antibodies used in this study.

Antibodies	Company (catalog no./ref)	Dilution used
Nipp1	Sigma (HPA027452)	IF 1:250; WB 1:1000
Gapdh	Cell Signalling (2118)	WB 1:5000
Sox9	Millipore (AB5535)	DAB 1:100; WB 1:1000
$p16^{INK4A}$	Cell Signalling (4824)	IF 1:50
Plzf	Santa Cruz (SC-28319)	IF 1:50; WB 1:1000
Cyclin D2	Cell Signalling (3741)	IF 1:75
BrdU	BD Biosciences (347580)	IF 1:100
H3S10P	Upstate (06-570)	IF 1:500
TBP	Abcam (ab51841)	WB 1:5000
PCNA	Upstate (07-2162)	IF 1:100; WB: 1:1000
GFRA1	R&D systems (AF560)	IF 1:50
SAP155	MBL (D221-3)	WB 1:2500
β-actin	Abcam (ab6276)	WB 1:2500
CDC5L	Home made [14]	WB 1:2500
FCM (PP1)	Home made [15]	WB 1:2500

Supplementary Table 4. qRT PCR primers used in this study

Gene	mRNA Forward primer	mRNA Reverse primer
Hprt	CTGGTGAAAAGGACCTCTCG	TGAAGTACATTATAGTCAAGGGCA
Ppp1r8 (Nipp1)	AGCGCTGGTGTACCACAAACA	TTGTGAGGTTCCAGCCGAATGT
Ddx4 (Vasa)	TGGCAGAGCGATTTCTTTT	CGCTGTATTCAACGTGTGCT
Plzf	GAGCACACTCAAGAGCCACA	GTGGCAGAGTTTGCACTCAA
Stra8	GCTTTTGACGTGGCAAGTTT	AACACAGCCAAGGCTTTTGA
Tacstd1	CAGAATACTGTCATTTGCTCCA	GTTCTGGATCGCCCCTTC
Sycp3	GGACAGCGACAGCTCACC	TTCCCAGATTTCCCAGAATG
Stmn1	CTGCAGAAGAAAGACGCAAGT	TGCTGAAGTTGTTGTTCTCCTC
Prm2	CAGAAGGCGGAGGAGACAC	CTCCTCCTTCGGGATCTTCT
Tpn1	AGCCGCAAGCTAAAGACTCA	CGGTAATTGCGACTTGCAT
Tnk1	CTCAAGTGTCTGATTCCAGAGG	CCACTGGGTAGTGTCCATAAC
Ptprt	TTACCTGGGAGCAGATTAACAC	CCAGAGCTGTTCACCATCAT
Dusp9	CTTGAGCTGTGGCCTAGATT	TAGGTAGAGATTGGGCAGGA
Nanos1	CTACACCACACACATCCTCAA	CTTTGGAGAGCGGGCAATA
Pou3f3	AGCAGTTCGCTAAGCAGTT	CGAGAACACGTTGCCATAGA
Lhx9	CCGAGACTCTGTCTACCATCT	GCTGTCCTTCATCCCGAAAT
Parp8	GGATCACTTCCGAAACCACTT	TCTCTCTTGCCTTGAACACATC
Bbc3	GTCTAGCCCGCGACAGT	CGCAAAGGCTGCAGGATAC
Cdkn2a	GAACTCTTTCGGTCGTACCC	GTTCGAATCTGCACCGTAGT
Ccnb3	GCTGGTGGAGACTGAAGATTAC	CTCCTCAATGATGGTTGGATCTT
Kntc1	TCCGAAGATCCAAGCATTCAG	GACCACATCCACGTCAGTATC
Kif2b	GGATCTGTGTGTGTGTGAGAA	ACTACATTGTGCGAGGGAATAG
Dnmt3l	GAGACACCTTCTTCTTGCTCTAA	GGATTTCAGCCATTGCTCTTC
Ggra1	AAGAGAAGAATTGTCTGCGTATCT	CTGCTGTTAACCGGCTCAT
Gdnf	GCTGACCAGTGACTCCAATATG	CGCTGCCGCTTGTTTATCT
Inhba	AGAACGGGTATGTGGAGATAGA	GACTCGGCAAAGGTGATGAT
Inha	TCTGAACCAGAGGAGGAAGAT	GGGATGGCCGGAATACATAAG
Shbg	CACAGTAGGCTTTGGTCCTC	CTCAGGCATAGCATCTCCTTC

Sox9	GAAGGAGAGCGAGGAAGATAAG	TGACGTGTGGCTTGTTCT
Gapdh	CCGCATCTTCTTGTGCAGTG	CCGTTGAATTTGCCGTGAGT
Itga6	GGACAACGTGATCCGGAAAT	CAACAGCCGCTTGTCCT
Itgb1	CTCCGGCCAGAAGACATTAC	GGGTAATCTTCAGCCCTCTTG

Supplementary references

1. Martin, M. Cutadapt removes adapter sequences from high-throughput sequencing reads. *EMBnet.journal* **17,** 10 (2011).
2. Morgan, M. *et al.* ShortRead: A bioconductor package for input, quality assessment and exploration of high-throughput sequence data. *Bioinformatics* **25,** 2607–2608 (2009).
3. Langmead, B. & Salzberg, S. L. Fast gapped-read alignment with Bowtie 2. *Nat Methods* **9,** 357–359 (2012).
4. Trapnell, C., Pachter, L. & Salzberg, S. L. TopHat: Discovering splice junctions with RNA-Seq. *Bioinformatics* **25,** 1105–1111 (2009).
5. Li, H. *et al.* The Sequence Alignment / Map (SAM) Format and SAMtools 1000 Genome Project Data Processing Subgroup. *Bioinformatics* **25,** 1–2 (2009).
6. Trapnell, C. *et al.* Transcript assembly and quantification by RNA-Seq reveals unannotated transcripts and isoform switching during cell differentiation. *Nat. Biotechnol.* **28,** 511–515 (2010).
7. Quinlan, A. R. & Hall, I. M. BEDTools: A flexible suite of utilities for comparing genomic features. *Bioinformatics* **26,** 841–842 (2010).
8. Anders, S., Pyl, P. T. & Huber, W. HTSeq-A Python framework to work with high-throughput sequencing data. *Bioinformatics* **31,** 166–169 (2015).
9. Risso, D., Schwartz, K., Sherlock, G. & Dudoit, S. GC-content normalization for RNA-Seq data. *BMC Bioinformatics* **12,** 480 (2011).
10. Robinson, M. D. & Smyth, G. K. Moderated statistical tests for assessing differences in tag abundance. *Bioinformatics* **23,** 2881–2887 (2007).
11. Benjamini, Y. & Hochberg, Y. Controlling the false discovery rate: a practical and powerful approach to multiple testing. *Journal of the Royal Statistical Society* **57,** 289–300 (1995).
12. Feil, S., Valtcheva, N. & Feil, R. Inducible cre mice. *Methods Mol. Biol.* **530,** 343–363 (2009).
13. Russell LD, Ettlin RA, SinhaHikim AP, C. E. *Histological and histopathological evaluation of the testis*. (Clear-water, FL: Cache River Press, 1990).
14. Boudrez, A. *et al.* NIPP1-mediated interaction of protein phosphatase-1 with CDC5L, a regulator of pre-mRNA splicing and mitotic entry. *J. Biol. Chem.* **275,** 25411–25417 (2000).
15. Van Dessel, N. *et al.* The phosphatase interactor NIPP1 regulates the occupancy of the histone methyltransferase EZH2 at Polycomb targets. *Nucleic Acids Res.* **38,** 7500–7512 (2010).
